# Multistrain Probiotics Plus Vitamin D Improve Gut Barrier Function and Gut Microbiota Composition in Irritable Bowel Syndrome Without Constipation: Results from a Double-Blind, Randomized, Placebo-Controlled Trial

**DOI:** 10.3390/nu17101708

**Published:** 2025-05-18

**Authors:** Lucrezia Laterza, Cesare Cremon, Gaetano Coppola, Carlo Romano Settanni, Rossella Maresca, Martina Strazzeri, Eleonora Durini, Valentina Petito, Franco Scaldaferri, Giorgio Gargari, Diego Mora, Elnaz Vojoudi Yazdi, Chiara Marangelo, Gianluca Ianiro, Lorenza Putignani, Maria Raffaella Barbaro, Giovanni Marasco, Giovanni Barbara, Antonio Gasbarrini

**Affiliations:** 1 CEMAD Digestive Disease Center, Fondazione Policlinico Universitario Agostino Gemelli IRCCS, 00168 Rome, Italy; lucrezia.laterza@policlinicogemelli.it (L.L.); rossella.maresca12@gmail.com (R.M.); martina.strazzeri@policlinicogemelli.it (M.S.); eleonora.durini@policlinicogemelli.it (E.D.); franco.scaldaferri@policlinicogemelli.it (F.S.); gianluca.ianiro@policlinicogemelli.it (G.I.); antonio.gasbarrini@unicatt.it (A.G.); 2 Dipartimento di Medicina e Chirurgia Traslazionale, Università Cattolica del Sacro Cuore, L.go F. Vito 1, 00168 Rome, Italy; 3 Department of Medical and Surgical Sciences, University of Bologna, 40100 Bologna, Italy; cesare.cremon@aosp.bo.it (C.C.); maria.barbaro2@unibo.it (M.R.B.); giovanni.marasco4@unibo.it (G.M.); giovanni.barbara@unibo.it (G.B.); 4 IRCCS Azienda Ospedaliero-Universitaria di Bologna, 40100 Bologna, Italy; 5 Unit of Gastroenterology and Digestive Endoscopy, Scientific Institute for Research, Hospitalization and Healthcare (IRCCS) Italian National Research Centres on Aging (INRCA), 60100 Ancona, Italy; c.settanni@inrca.it; 6 CeMAD Translational Research Laboratories, Digestive Disease Center (CeMAD), Department of Medical and Surgical Sciences, Fondazione Policlinico Universitario “A. Gemelli” IRCCS, 00146 Rome, Italy; valentina.petito@policlinicogemelli.it; 7 Department of Food, Environmental and Nutritional Sciences (DeFENS), University of Milan, 20121 Milan, Italy; giorgio.gargari@unimi.it (G.G.); diego.mora@unimi.it (D.M.); elnazvojoudiyazdi@unimi.it (E.V.Y.); 8 Unit of Microbiome, Bambino Gesù Children’s Hospital, IRCCS, 00146 Rome, Italy; chiara.marangelo@opbg.net; 9 Unit of Microbiomics and Unit of Microbiome, Bambino Gesù Children’s Hospital, IRCCS, 00146 Rome, Italy; lorenza.putignani@opbg.net; 10 Department of Life Science, Health, and Health Professions, Link Campus University, 00165 Rome, Italy

**Keywords:** IBS, probiotic, gut barrier

## Abstract

Background: The disruption of the intestinal barrier and the imbalance of the gut microbiota (GM) seem to play a major role in the complex pathogenesis of irritable bowel syndrome (IBS). Specific microbial strains could improve the gut microenvironment, promoting anti-inflammatory pathways; similarly, vitamin D supplementation could play a role in enhancing the barrier integrity and modulating the immune response in the gut. This study aims to evaluate the efficacy of a new multistrain probiotic, combined with vitamin D, in improving gut barrier function in IBS without constipation. Methods: In this phase IIb double-blind randomized placebo-controlled, parallel-group, multicenter, clinical trial, 35 patients were treated for 12 weeks with OttaBac^®^, a high concentration multistrain probiotic plus cholecalciferol, or placebo and were followed up until week 16. Symptoms, quality of life, intestinal permeability, fecal biomarkers, and microbiota composition were evaluated at 0, 12, and 16 weeks. Results: Mean zonulin values showed a significant progressive reduction in the active group (−10.2 ng/mL at week 12, *p* = 0.0375; −19.5 ng/mL at week 16, *p* = 0.0002), with a significant difference between groups at week 16 in the per-protocol population (−19.01, *p* = 0.0053). The active group showed a more stable trend toward improvement in stool frequency and consistency at both week 12 and 16, with a significant improvement compared to the baseline and to the placebo group (−23.2, *p* = 0.0265, and 5.57 vs. −23.2, *p* = 0.0492, respectively). No differences were found in regards to the lactulose/mannitol ratio, Irritable Bowel Syndrome Severity Scoring System (IBS-SSS) and Short Form Health Survey (SF-36) total scores, plasmalemmal vesicle associated protein-1 (PV-1), and citrulline levels. In the active group, *Bifidobacterium animalis* subsp. *lactis* and *Streptococcus thermophilus* levels were increased (*p* < 0.05), while those for *Lachnospira* were decreased (*p* < 0.05), and significant changes in Actinobacteria and Proteobacteria were observed (*p* < 0.05). Lactate (*p* < 0.01) and acetate (*p* < 0.05) levels increased post-treatment. Correlation analysis pointed out a significant association between the microbial biomarkers and the symptoms (*p* < 0.05). Conclusions: Probiotic plus vitamin D could improve IBS-associated symptoms through gut microbiota modulation and gut barrier enhancement, with persistent benefits after treatment discontinuation.

## 1. Introduction

Irritable bowel syndrome (IBS) is a common functional gastrointestinal disorder. The pathogenesis of IBS is still not completely clear; however, increasing evidence suggests that dysbiosis and gut barrier dysfunction could play a major role in this condition. Particularly, in diarrhea-predominant IBS (IBS-D), epithelial tight junction impairment with reduced epithelial resistance could have a key pathogenetic role [1].

However, intestinal permeability is not easily investigated, as non-invasive biomarkers and tools display several limitations related to the lack of validation and standardization; for this reason, combining more than one marker has been proposed to better assess intestinal permeability in clinical trials [2]. Considering the multiple tests available for the assessment of intestinal permeability, the urinary lactulose/mannitol (LaMa) test, as well as zonulin, citrulline, and plasmalemmal vesicle associated protein-1 (PV-1) serum level assessment are among those most widely used.

The LaMa test is a widely used test employed to evaluate intestinal permeability due to the different characteristics of both involved sugars. Mannitol is a small molecule that can permeate the intestinal barrier through both the paracellular and transcellular pathway; lactulose, instead, is too large to permeate the small transcellular pathways and selectively permeates through the paracellular passageways. Considering that mannitol exhibits few paracellular compared to transcellular pathways, it could effectively measure the transcellular pathways; moreover, its permeation rate is directly related to the intestinal surface area. Instead, lactulose permeates only the damaged area on the same intestinal surface. Finally, the ratio of LaMa excretion in urine provides an indirect estimation of intestinal permeability, and its increase correlates with an increase in overall intestinal permeability [3].

Zonulin is the endogenous counterpart of *Vibrio cholera* enterotoxin, and currently, it is the only known human protein which can reversibly open tight junctions. An increase in zonulin expression has been related to an increase in intestinal permeability and could be considered a reliable marker in several gastrointestinal disorders, including inflammatory bowel disease, IBS, and celiac disease [4,5,6]. Citrulline may be considered as a biomarker of functional enterocyte mass, and it has been linked with enterocyte dysfunction, systemic inflammation, bacterial translocation, and clinical intestinal dysfunction [7]. However, it can also be considered as a marker of bacterial active metabolism, with reduced levels after probiotic supplementation [8]. PV-1 is a marker of endothelial permeability, and increased serum levels may be detected in patients with celiac disease and concomitant liver injury [9]. Vitamin D could exert pleiotropic effects; it is specifically known for its effect on the innate and adaptive immune systems [10], and it could be helpful in the treatment of IBS, based on its gut microbiota modulation, immune regulation, and inflammation reducing properties [11].

In IBS, gut barrier could be a potential therapeutic target, also through its influence on gut microbiota (GM) composition. GM modulation could be performed in multiple ways, using diet, prebiotics, and antibiotics, but probiotics constitute one of the most interesting options, based on their potential pleiotropic effects and their long-term safety. Evidence supporting the efficacy of probiotics in treating IBS is still controversial, even if each probiotic strain could have a theoretically positive effect on gut barrier function, enhancing the mucus layer, increasing tight junction expression, interacting with microbes in the host, and regulating the immune cells in the lamina propria. However, not all probiotic strains show all of these effects at the same time, as some mechanisms are considered species-specific and even strain-specific [12]; moreover, multistrain compared to single-strain probiotics could be more effective in treating several gut-related diseases, despite evidence showing no difference between single strains vs. mixtures in selected cases [13]. Multistrain probiotic products could combine different mechanisms of action and increase the efficacy of modulation through a synergistic effect among the strains. Similarly, vitamin D could play a role in IBS pathogenesis; vitamin D receptor (VDR) is strongly expressed in the gut, where vitamin D/VDR signaling could play a beneficial role in modulating the mucus layer, the immune response, and the composition of the microbiota in the gut [14,15]. Vitamin D promotes Treg immune response and influences the expression of tight junction proteins, enhancing the integrity of the intestinal barrier, consequently influencing the gut microenvironment in the anti-inflammatory pathways and indirectly modifying the GM composition. However, the dynamic interplay between the intestinal barrier, GM, and vitamin D remains difficult to untangle, as the GM can modulate vitamin D metabolism and receptor expression through a complex, bidirectional relationship not yet adequately explored [14]. Low levels of vitamin D have been associated with IBS, as its mean values are lower in IBS patients compared to in control subjects [16]. Based on this background, a new product composed of a highly concentrated multistrain probiotic consortium (OttaBac^®^) and vitamin D has been developed. OttaBac^®^ has been evaluated in a murine model of stress-related mood disorders, based on maternal separation, and has been demonstrated to reverse the anxiety- and depressive-like behavior, normalize the neuro-inflammatory state by restoring the resting state of the microglia, and finally, inducing a pro-neurogenic effect [17]. Similarly, in a mouse model of acute inflammation induced by an intraperitoneal single injection of lipopolysaccharides (LPS), OttaBac^®^ administration prevented the LPS-dependent increase in pro-inflammatory cytokines in the gastrointestinal tract and the brain and enhanced gut barrier function through increased epithelial junction expression in the colon [18]. The aim of this pilot study is to evaluate the synergistic effect of this consortium on the pathophysiological, symptomatic, and quality of life-related aspects in patients with IBS without constipation.

## 2. Materials and Methods

**Figure 1:**
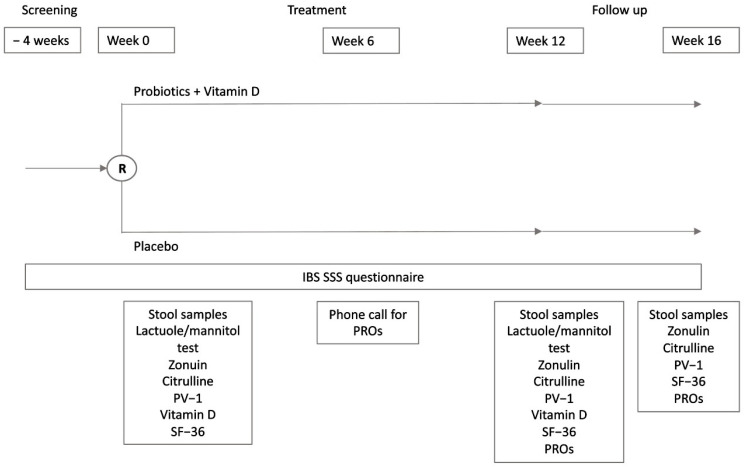
Schematic representation of study design. PROs, patient reported outcomes; PV-1, plasmalemma vesicle associated protein-1; SF-36, Short Form Health Survey.

This is a phase IIb, double-blind, randomized, placebo-controlled, parallel-group, multicenter, competitive trial. The study included a 4-week screening phase (V1), a baseline visit (V2), a 6-week intermediate phone visit (V3), a 12-week treatment phase (V4), and a further 4-week follow-up visit at week 16 (V5) (Figure 1). After the informed consent form was signed, patients had up to 4 weeks to be randomized 1:1 to receive one sachet per day of the active product or a matching placebo for 12 weeks. During the screening phase, data regarding medical history were collected, inclusion and exclusion criteria were checked, and an IBS-SSS questionnaire was given to the patients. The patients were trained to fill out one IBS-SSS questionnaire every week and one Bristol stool scale questionnaire every day. At baseline, eligible patients were asked to provide a stool sample for gut microbiome and short chain fatty acids (SCFAs) analysis, a blood sample for serum biomarker and vitamin D plasma level assessment, and a urine sample for LaMa testing, and they were required to complete the SF-36 questionnaire. They were then randomized to receive the treatment or placebo. The randomization list, with blocks (block size = 4), was computer-generated by an independent statistician not involved in data collection and analysis, and this information was known only by the CRO. Investigators and patients were blind to the randomization list. At the time of randomization, the centers assigned the investigational product (IP) kit to the patient with the lowest available number and contacted the CRO for confirmation. After 6 weeks of treatment, investigators checked for patient-reported outcomes, eventual adverse events (AEs), and concomitant medication use through a phone call. At week 12, patients came back to the centers for a new clinical evaluation and to provide fecal, blood, and urine samples for gut microbiome and SCFAs analysis, serum biomarker and vitamin D plasma level analysis, and LaMa testing, respectively. Quality of life and patient reported outcomes were also re-evaluated at the end of treatment. A total of 4 weeks after treatment discontinuation (week 16), patients were clinically evaluated for IBS symptoms and IBS-related quality of life (follow-up). During the follow-up visit, patients also provided a blood sample for biomarker assessment and a fecal sample for gut microbiome and SCFAs analysis. AEs were constantly monitored during the whole trial duration.

### 2.1. Study Population

We conducted this study on a population of IBS non-constipation adults, defined according to the Rome IV criteria [19], aged 18–65 years, who were followed-up in two referral centers in Italy: Fondazione Policlinico Universitario A. Gemelli IRCCS in Rome and IRCCS Azienda Ospedaliero Universitaria di Bologna in Bologna. Patients were required to conform to the study protocol, to provide their free and informed consent, and to maintain a stable diet two months before the screening visit and during the study. Exclusion criteria were IBS with predominant constipation (IBS-C) or IBS unclassified (IBS-U), according to the Rome IV criteria. Exclusion criteria: patients with any relevant organic, systemic, or metabolic disease (specifically, a significant history of cardiac, renal, neurologic, oncologic, endocrinologic, metabolic, or hepatic disease), ascertained unstable psychiatric conditions, or with ascertained intestinal organic diseases; previous major abdominal surgeries (except for previous appendectomy); use of anti-secretory drugs, including all proton pump inhibitors and H2-antagonists, during the preceding two months before screening; introduction or changes in dose of other drugs influencing intestinal permeability during the last 4 weeks before screening; assumption of probiotics or topic and/or systemic antibiotic therapy during the previous 2 months; and pregnancy. This study was conducted according to the principles of the Declaration Helsinki and good clinical practice, after receiving IEC approval from both centers (Protocol 41259/19 ID:2817, meeting 24 October 2019; 25/2020/Sper/AOUBo, meeting 23 January 2020).

### 2.2. Study Products

The IP is a combination of a probiotic consortium (OttaBac^®^) at a high concentration (500 billion CFU/sachet) and vitamin D (2000 UI of cholecalciferol/sachet). OttaBac^®^ is a probiotic mixture containing eight live, freeze-dried bacterial strains: *Bifidobacterium animalis* subsp. *lactis* BL03, *Streptococcus thermophilus* BT01, *B. animalis* subsp. *lactis* BI04, *Lactiplantibacillus plantarum* BP06, *Lactobacillus acidophilus* BA05, *Lacticaseibacillus paracasei* BP07, *Lactobacillus helveticus* BD08, and *Bifidobacterium breve* BB02 [18]. Patients were instructed to take the product with water on an empty stomach. The placebo contained maltose and cornstarch. The active product and the placebo were packed in equal sachets and were indistinguishable, based on organoleptic properties.

### 2.3. Study Outcomes

The aims of the study were to evaluate the effect of the IP on the in vivo intestinal permeability-assessed measurement of urinary excretion of orally administered lactulose and mannitol; to evaluate the effect of IP on gut barrier function assessed by serum biomarkers, including zonulin, citrulline, and PV-1; to evaluate the effect of the IP on GM ecology and function assessed through 16S-RNA characterization, PICRUSt-based functional pathway prediction, and metabolic activity based on fecal concentration of SCFAs (acetate, butyrate, propionate); to evaluate the effect of the IP on IBS symptoms through the evaluation of variations in responses to the IBS-SSS questionnaire and Bristol stool scale; to evaluate the effect of the IP on quality of life through SF-36; and to evaluate the effect on IP on serum levels of vitamin D.

### 2.4. Statistical Analysis

This is a pathophysiological study, and no power calculations were performed to identify the number of patients required to detect statistical significance. Indeed, there are no previously conducted reference studies to which the statistical power could be referred. However, we planned to include 48 patients in the study, based on feasibility criteria and previously published studies with similar pathophysiological endpoints [20]. The study population for the analysis of the efficacy variables (intention-to-treat analysis) comprised all patients who received at least one dose of trial treatment and completed at least one post-baseline diary assessment. In the analysis outcome of the intent-to-treat population (ITT), when there were more than two time points, missing data were not replaced but were interpolated by mixed models. When there were only two time points, the last observations carried forward (LOCF) were used to impute data. In addition, a separate per-protocol analysis was performed. The weekly frequency and stool consistency assessments recorded using the Bristol Stool Form Scale were calculated as the mean of the daily data. The baseline for diary data was the 2-week daily average (on available data), before randomization. Bowel movements were considered normal if the Bristol stool scale was <5 and the frequency of bowel movements was ≤3/day; otherwise, they were considered “abnormal”. The percentage of days in a week with abnormal bowel movements was also calculated. Frequencies, means, standard deviations, and ranges were used as descriptive statistics. For categorical variables, the absolute counts (n) and percentages (%) were presented for each category. The Yates’ corrected X-square, the Mann–Whitney, and the Wilcoxon tests were applied, as appropriate. The odds ratio, together with the 95% confidence interval, were used to compare the response between the two treatments. For continuous data, analysis of covariance was used, including factors regarding study group and baseline value, to evaluate differences among groups. A two-tailed *p*-value less than 0.05 was considered statistically significant. Data were analyzed using the SPSS/PC+ statistical package.

### 2.5. Lactulose/Mannitol Test

Urinary LaMa samples from all the centers were shipped to a central lab, and all testing was performed at the same laboratory (CDI Centro Diagnostico Italiano, Milan, Italy) in charge of performing mass spectrometry for the quantification of urinary concentrations of the two sugars. At screening, patients were provided with a urinary container and were instructed about the correct protocol for urinary sample collection. At baseline and on week 12, before coming to the center, patients collected their first morning urine, adding chlorhexidine to the sample in the first container. At the center, patients were administered a LaMa syrup and were instructed to collect all their urine for the next 6 h in a second container, adding chlorhexidine and returning the sample to the center at the end of the collection period. From the two containers, two tubes of urine were extracted, respectively, and stored at +2/+8 °C before being shipped and analyzed within 2 weeks from the time of collection. A ratio of LaMa ≥ 0.03 was considered consistent with hyperpermeability.

### 2.6. Serum Biomarkers and Vitamin D Levels

The following serum biomarkers were evaluated: zonulin, citrulline, and PV-1. Serum biomarkers (namely zonulin, citrulline, and PV-1) were evaluated at baseline, at week 12, and at week 16, whereas vitamin D levels were evaluated only at baseline and week 12. Blood samples were collected by venipuncture and centrifuged at 2000× *g* for 10 min at 18–25 °C to separate the serum. The serum was stored at −80 °C for zonulin, citrulline, and PV-1 and at −20 °C for vitamin D, respectively, up to the time of shipment, and the samples were analyzed within 2 months after collection. The serum biomarkers were centrally shipped and analyzed at the same laboratory (CDI Centro Diagnostico Italiano, Milan, Italy). The ELISA test was employed for zonulin, citrulline, and PV-1. The 25-hydroxycholecalciferol levels were measured in the serum. Normal values for zonulin were between 20 and 48 ng/mL.

### 2.7. Ecological and Functional Characterization of Gut Microbiota and SCFAs Analysis

Fecal samples were collected at baseline (V2), week 12 (V4), and week 16 (V5), with a tolerance of ± 4 days, for metataxonomic analysis targeting the 16S rRNA V3-V4 gene portion to profile the microbial communities. These samples were stored at −80 °C until DNA extraction was performed in the centers for the duration of the trial. Once all the stool samples were collected, they were shipped under refrigeration to the centralized laboratory (DEFENS) in charge of performing GM profiling and SCFA analyses. For the extraction process, the fecal samples were thawed at +4 °C and vigorously mixed for 2–3 min with a sterile spatula. Subsequently, 150 mg of feces were weighed and processed using the PowerSoil Pro Kit (Qiagen, Germantown, USA), according to the manufacturer’s instructions. The extracted DNA was quantified using the Qubit Broad Range kit (Thermo Fisher Scientific, Waltham, MA, USA) and used for metataxonomic analysis through 16S rRNA gene profiling. The NovaSeq 6000 platform with 2 × 250 bp sequencing (NovaSeq 6000 SP Reagent Kit, 5 Gbases, Illumina, Cambridge, UK) was used to sequence the 16S rRNA gene amplicons, targeting the V3 and V4 variable regions. These amplicons were obtained using primers 341F (5′-CCTACGGGNBGCASCAG-3′) and 805R (5′-GACTACNVGGGTATCTAATCC-3′) (BMR Genomics, Padua, Italy). The sequencing reads were processed using the Quantitative Insights Into Microbial Ecology 2 (QIIME2), version 2023.2, bioinformatics pipeline, employing the divisive amplicon denoising algorithm (DADA2) [21]. The Greengenes Database, version 13_8, was used for taxonomic assignment to amplicon sequence variants (ASVs). To minimize technological bias, the 16S rRNA gene profiling analysis was conducted simultaneously for all fecal samples in this study. A mock community from BMR Genomics was inserted between the samples to validate the output after processing the reads. Metataxonomic raw sequencing data are available as FASTQ files in the European Nucleotide Archive (ENA) of the European Bioinformatics Institute under the accession code PRJEB85279.

#### 2.7.1. Quantification of SCFAs

The quantification of SCFAs in fecal samples was carried out following the method described by Gargari et al. [22]. Briefly, 100 mg of stool was suspended in 2 mL of 0.001% formic acid, vortexed for 1 min, and centrifuged at 1000× *g* for 2 min at 4 °C. The supernatant was recovered, and the pellet was re-extracted following the same procedure. The two supernatants were combined, and the volume was adjusted to 5 mL with 0.001% formic acid solution. All extracts were stored at −20 °C until analysis. The analysis was performed using UPLC-HR-MS on an Acquity UPLC separation module (Waters, Milford, MA, USA), coupled with an Exactive Orbitrap MS via a HESI-II probe for electrospray ionization (Thermo Scientific, San Jose, CA, USA). The conditions for the column, ion source, and interface were the same as those reported by Gargari et al. [22]. Elution was conducted at a flow rate of 0.2 mL/min, with the following solvents: 0.001% HCOOH in MilliQ-treated water (solvent A) and CH3OH (1:1 *v*/*v*, solvent B). The elution gradient was as follows: 0% B for 4 min, 0–15% B over 6 min, 15–20% B over 5 min, and 20% B for 13 min, followed by a return to the initial conditions in 1 min. The UPLC eluate was analyzed using full scan MS over the range of 50–130 m/z, as detailed by Gargari et al. [22]. External calibration curves were created using reagents from Sigma-Aldrich (Milan, Italy) to quantify acetic, butyric, isobutyric, isovaleric, propionic, and valeric acids in the fecal samples. SCFA concentrations were expressed in mmol per kilogram of wet feces.

#### 2.7.2. Statistical Analysis of the Microbiome

The R programming language, version 4.3.1, was used in order to comprehensively compare the GM and SCFAs between the active and placebo groups at different time points (V2, V4, V5). Alpha diversity measures the richness and evenness within a sample. In our study, we used QIIME2 [23] to compute four indices: observed features (richness), Shannon’s entropy (richness and evenness), Pielou’s index (evenness), and Faith’s phylogenetic diversity (richness and evolutionary diversity). Beta diversity, which assesses differences between groups, was analyzed using ANOSIM [24] at weighted and unweighted UniFrac distances to incorporate phylogenetic relationships [25]. Various statistical methods were employed to analyze the microbiome data. PLSDA and sparse PLSDA (sPLSDA) were applied with CLR-transformed taxonomic data to distinguish between the active treatment and placebo groups, with ROC curves and confusion matrices used to validate model performance [26]. The Shapiro–Francia test assessed data normality, followed by Wilcoxon signed-rank or paired T-tests for paired samples, and Mann–Whitney or t-tests for unpaired data. SCFA levels were compared across time points. LEfSe analysis [27] identified microbial biomarkers and shifts in SCFAs between groups over time. Kendall correlation was used to assess relationships between gut bacteria, clinical parameters, SCFAs, and alpha diversity. Random forest analysis further validated the results by constructing multiple decision trees to improve prediction accuracy. It was performed using the “random forest” library in R (available at http://www.stat.berkeley.edu/users/breiman/, accessed on with access on 9 October 2024).

#### 2.7.3. Gut Microbiota Functional Pathway Prediction

Based on the KEGG Orthology (KO) database [23], the Phylogenetic Investigation of Communities by Reconstruction of Unobserved States of Correlation 2 (PICRUSt2 v2.5.2) software was used to predict the functional pathways [24]. To identify statistically significantly (*p*-value ≤ 0.05) different KO pathways between the active and placebo groups, at each V2, V4, and V5 time point, the Mann Whitney test was applied. Moreover, to evaluate the direction of each KO pathway, the ratio of the mean abundance of active and placebo groups was calculated by considering only the “unique” KO associated to each time point.

### 2.8. Clinical Evaluation

Subsequent to the screening visit and throughout the study, patients recorded data related to their daily bowel habits (frequency and stool consistency as assessed by the Bristol Stool Form Scale) and weekly abdominal pain, bloating, and general well-being, as assessed by a weekly 11-point numeric rating scale from “0” (none) to “10” (very severe), using a paper diary. To evaluate patient-reported outcomes (PROs), patients were asked to answer a “yes” or “no” question about their impressions regarding the efficacy of the treatment. Particularly, the following question: “Do you think that this treatment significantly improved your IBS symptoms?” was asked during V3 (by phone) and V4. Furthermore, patients were asked to complete an IBS-SSS [25] questionnaire on a weekly basis.

### 2.9. Quality of Life Evaluation

At baseline, at the end of the treatment period, and at the end of follow-up, quality of life was evaluated through a Short-Form 36 Items Health Survey (SF-36) [26].

## 3. Results

**Figure 2:**
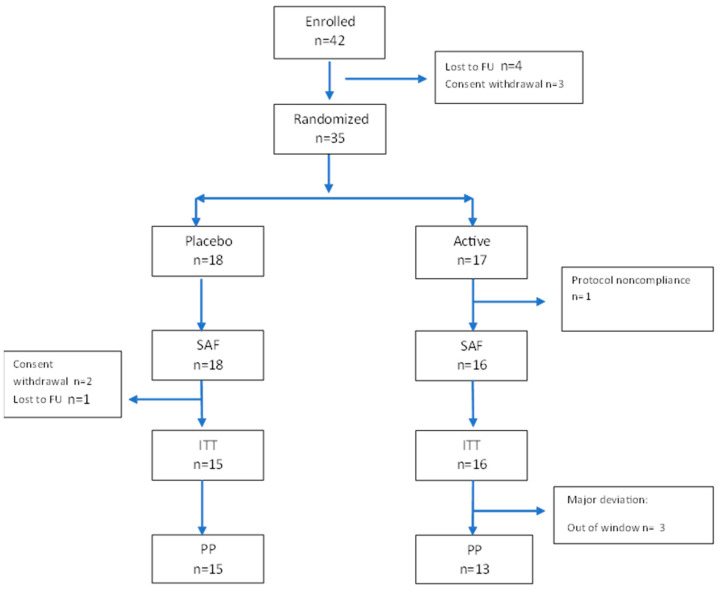
Participant flow diagram according to CONSORT guidelines. A total of 42 patients were enrolled in the study, 3 withdrew their consent, 4 discontinued the study before the randomization, 1 did not take any medication and was excluded from the SAF population, and 3 did not have any data after baseline and were excluded from the intent-to-treat set. One patient discontinued the study/treatment after day 3, and two finished the study before the end. Twenty-eight patients were included in the per protocol set. SAF, safety population; ITT, intent-to-treat population; PP, per-protocol population.

A total of 42 patients were enrolled in the study, and 35 were randomized. Figure 2 describes the patient flow. Table 1 describes the patient characteristics, which were equally distributed, without significant differences between the two groups at baseline. The treatment was generally well tolerated. Five patients (three in the placebo group and two in the active group) experienced at least one AE (15%, CI95% 4.95–31.1%), for a total of six AEs. No serious AEs (SAEs) were registered. The reported AEs in included migraine, COVID-19, and flu syndrome in the placebo group, and suspected oxyuriasis and upper abdominal pain (two times in the same patient) in the active group.

### 3.1. Gut Barrier Dysfunction and Intestinal Permeability

**Figure 3:**
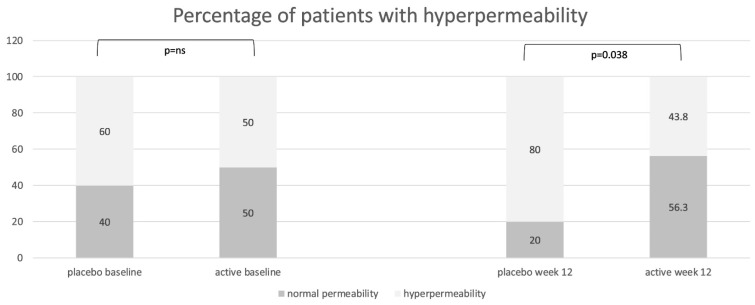
Distribution of patients with normal intestinal permeability (lactulose/mannitol ratio < 0.03) and with increased permeability (lactulose/mannitol ratio ≥ 0.03) in the placebo group and in the active group at baseline and at the end of treatment (week 12), expressed as percentages.

LaMa results at baseline were available for 31 patients. There was no significant difference between the two groups in either the intent-to-treat (ITT) or per-protocol (PP) set in regards to mean LaMa ratio baseline values: the placebo group and the active group showed a mean LaMa ratio of 0.08 (SD 0.074) vs. 0.05 (SD 0.035) in the ITT population and 0.08 (SD 0.077) vs. 0.05 (SD 0.035) in the PP population, respectively. From baseline to week 12, there were no significant variations in mean LaMa ratio between groups nor within groups in either the ITT or PP set. In fact, at week 12 mean, the LaMa ratio in the placebo and active groups was 0.08 (SD 0.068) vs. 0.05 (SD 0.028) for ITT and 0.08 (SD 0.070) vs. 0.05 (SD 0.028) for PP. Only 55% (n = 17/31) of patients showed an increased LaMa ratio at baseline, with no significant difference between distribution in the placebo or active groups (Figure 3). However, after treatment, a significant increase in the percentage of patients with high permeability was observed in the placebo group, but not in the active group. There was a trend in the active group showing a normalization of LaMa ratio. In fact, in the ITT population, in the active group, 25% of patients showed a normalization of the intestinal permeability, 56.3% showed no change compared to baseline, and 18.8% showed an increased intestinal permeability after treatment, notwithstanding normal values at baseline. In the placebo group, only 6.7% of patients showed normalization of a previously increased intestinal permeability, 66.7% showed no change, and 26.7% worsened after treatment (showing normal permeability at baseline and increased permeability at week 12). The same trend was confirmed in the PP population but without reaching statistical significance between groups: comparing the placebo group and the active group, 42.9% vs. 38.5% of patients showed a normal LaMa ratio at baseline, and 21.4% vs. 53.8% at week 12, respectively. After treatment, 64.3% vs. 53.8% showed no change in permeability, 7.1% vs. 30.8% showed normalization, and 28.6% vs. 15.4% showed a worsening in the placebo group and the active group, respectively.

**Figure 4:**
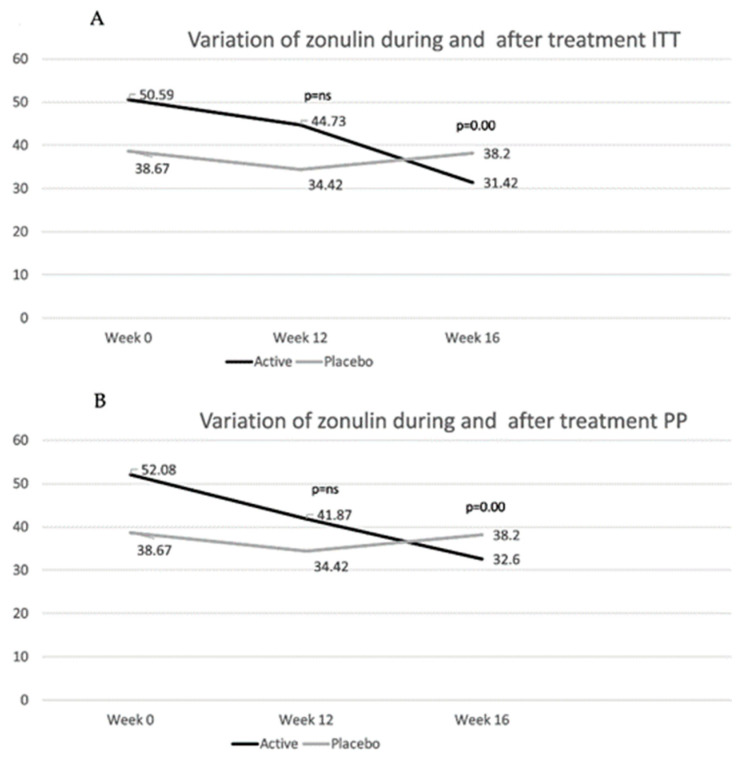
Mean values of zonulin in the intent-to-treat (ITT) (**A**) and in the per-protocol (PP) (**B**) population are shown. *p*-values refer to the differences between the active and the placebo group in the mean difference in variation in zonulin levels compared to those at baseline. (See text for *p*-values for mean difference of variation within groups before and after treatment.)

Only 12 out of 31 patients showed increased values of zonulin (>48 ng/mL) at baseline. Mean zonulin values showed a progressive reduction in the active group, whereas stable values were observed in the placebo group (Figure 4). In the ITT population, patients taking the placebo showed a mean variation of −4.25 at week 12 and −0.47 at week 16 (*p* = ns). In contrast, in the active group, at week 12, patients reached a mean variation of −5.86 (*p* = ns) and at week 16 of −19.2 (*p* < 0.0001, referring to within-group variation compared to baseline). At week 16, the mean difference in variation compared to baseline was significantly higher in the active group compared to that in the placebo group (*p* = 0.0046). In the PP population, at week 12, patients in the active group showed a −10.2 reduction in zonulin compared to baseline levels (*p* = 0.0375) vs. −4.25 in the placebo group (*p* = ns). At week 16, zonulin further reduced in the active group (−19.5, *p* = 0.0002 compared to baseline), whereas it tended to return to baseline values in the placebo group (−0.47, *p* = ns compared to baseline). The mean variation difference between the two groups reached statistical significance at week 16 (−19,01, *p* = 0.0053). Comparing patients with increased LaMa ratio at baseline with patients with normal values, irrespective of the randomization groups, no differences in mean values between these two groups were found (47.35 ng/mL vs. 39.31 ng/mL in patients with a normal vs. a high LaMa ratio, respectively), showing a poor correlation between LaMa ratio and serum zonulin.

No significant differences were found before and after treatment or between groups in regards to citrulline and PV-1 variation; specifically, in the ITT population, the mean values of citrulline were 12.21 nmol/L at week 0, 13.29 nmol/L at week 12, and 13.86 nmol/L at week 16 in the placebo group; 13.20 nmol/L at week 0, 11.66 nmol/L at week 12, and 13.31 nmol/L at week 16 in the active group. Similarly, in the PP population, the citrulline mean values were 12.21 nmol/L at week 0, 13.29 nmol/L at week 12, and 13.86 nmol/L at week 16 in the placebo group; 14.26 nmol/L at week 0, 12.01 nmol/L at week 12, and 14.14 nmol/L at week 16 in the active group. For PV-1, in the ITT population, the mean values were 7.98 ng/mL at week 0, 6.21 ng/mL at week 12, and 5.69 ng/mL at week 16 in the placebo group; 5.98 ng/mL at week 0, 6.04 ng/mL at week 12, and 4.81 ng/mL at week 16 in the active group. In the PP population, they were 7.98 ng/mL at week 0, 6.21 ng/mL at week 12, and 5.69 ng/mL at week 16 in the placebo group; 5.31 ng/mL at week 0, 5.20 ng/mL at week 12, and 4.02 ng/mL at week 16 in the active group.

### 3.2. Vitamin D

The vitamin D mean levels were similar in the active and placebo group at baseline (ITT 29.51 ± 11.08 vs. 22.85 ± 7.03 ng/mL; PP 28.85 ± 11.10 vs. 22.85 ± 7.03 ng/mL in the active and placebo groups, respectively), and they significantly increased in the active group after 12 weeks of treatment (ITT 43.88 ± 13.47 vs. 25.73 ± 8.62 ng/mL, *p* = 0.0021; PP 43.13 ± 14.57 vs. 25.73 ± 8.62 ng/mL, *p* = 0.0054 for the active and placebo groups, respectively).

### 3.3. Clinical Outcomes

Both groups showed a significant reduction in IBS-SSS at week 16, without significant differences between the active and the placebo groups (Supplementary Table S1 shows results about single items for the IBS-SSS in the ITT and PP populations). No significant differences were found between groups in regards to the variation between the Bristol stool scale mean values. In the ITT population at baseline, the mean Bristol stool scale values were 4.14 vs. 4.83 in the active and placebo groups, respectively, without significant variations in either group compared to the baseline regarding the subsequent time points (Supplementary Tables S2 and S3 show the distribution of patients in different categories of stool consistency according to the Bristol stool scale in the ITT and PP populations, respectively: 1–2 is considered as constipation, 3–5 as normal, and 6–7 as diarrhea).

**Figure 5:**
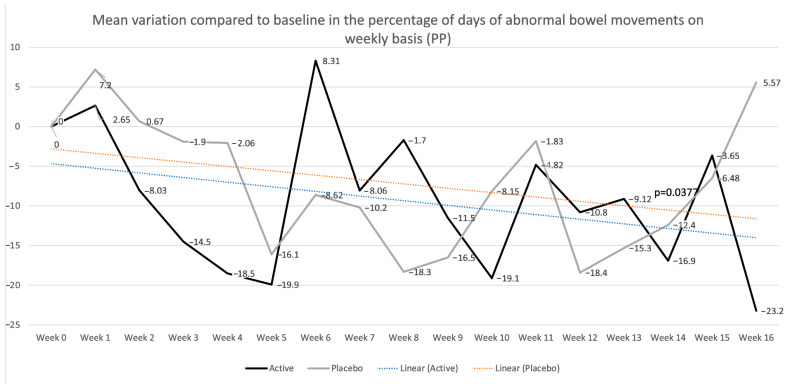
Mean percentages of days of abnormal bowel movements on a weekly basis in the per-protocol (PP) population are shown. *p* values refer to the mean differences in variation between the placebo and active groups (−31.82, *p* = 0.0377) at week 16.

Furthermore, when evaluating the percentage of days of abnormal bowel movements, where a day with normal bowel movements is defined as a day with less than or equal to three bowel movements per day with normal stool consistency (Bristol stool scale 3–5), in the PP population, patients in the placebo group showed a trend toward improvement during treatment but without significant results and an increase in abnormal days at week 16, which was numerically higher when compared to baseline levels (Figure 5). Patients in the active group showed a more stable trend toward improvement, maintaining this trend after treatment discontinuation, showing significant improvement compared to baseline at week 16 (−23.2, *p* = 0.0265), with significant differences compared to the results for the placebo group at week 16 (mean variation compared to baseline 5.57 vs. −23.2, *p* = 0.0492). These trends were also observed in the ITT population, with a non-significant improvement up to week 15 and a worsening at week 16, with a higher percentage of abnormal bowel movements compared to baseline levels (5.52) in the placebo group, whereas the active population showed a stable trend toward improvement, reaching statistical significance at week 16 (−25.1) compared to baseline levels and between the two groups (*p* = 0.0289). Considering the PROs, the percentage of patients answering yes to the question “Do you think that this treatment significantly improved your IBS symptoms?” was similar in both groups, in both the ITT and PP populations. Particularly, in the ITT population, 60% of patients in the placebo group were satisfied with their treatment at week 6, compared to 43.8% in the active group. At week 12, 66.7% of patients in the placebo group were satisfied compared to 60% in the active group.

### 3.4. Quality of Life

At baseline, there was no significant difference between groups in the different domains evaluated by SF-36. At week 16, the change in the social functioning score was significantly higher in the active group compared to that in the placebo group (in the ITT but not in the PP set), with a mean increase of 20 (5.99) points in the active group and 0.83 (5.99) in the placebo group (*p* = 0.028) (Table 2).

### 3.5. Gut Microbiota—Ecological and Functional Analyses

GM analyses were conducted on 27 patients (PP set) included in this analysis (15 in the placebo group and 12 in the active probiotic group). Alpha and beta diversity analyses were conducted. The UniFrac analysis did not reveal any clustering of the subjects. As for alpha diversity, no significant differences were found between the time points for either treatment group. This suggests that the microbial diversity remained stable over time within each group.

A statistical paired test was performed to compare the GM modulation between the active and placebo groups across all time points (V2, V4, V5). We observed a modulation of different bacteria after the intake of the active treatment compared to the placebo (Supplementary Figure S1). Notably, there was an increase in two specific bacteria in the active formulation group, i.e., *Bifidobacterium animalis* subsp. *lactis* and *Streptococcus thermophilus*. Additionally, several species within the Lachnospiraceae family were found to be more abundant after the active treatment compared to the levels in the placebo group. However, *Lachnospira* showed decreased levels in subjects treated with the active formulation compared to the levels in those receiving the placebo. Moreover, a single species from the genus *Dorea* increased in the group receiving the active product, whereas the genus *Dorea* showed a significant increase after the placebo intake. Interestingly, a species belonging to Ruminococcaceae increased after the active treatment, showing an inverse trend with that for the placebo group. Focusing on SCFAs, we observed a lactic acid increase after both the active and placebo treatment and an increase in acetate only after the placebo treatment (Supplementary Figure S2).

**Figure 6:**
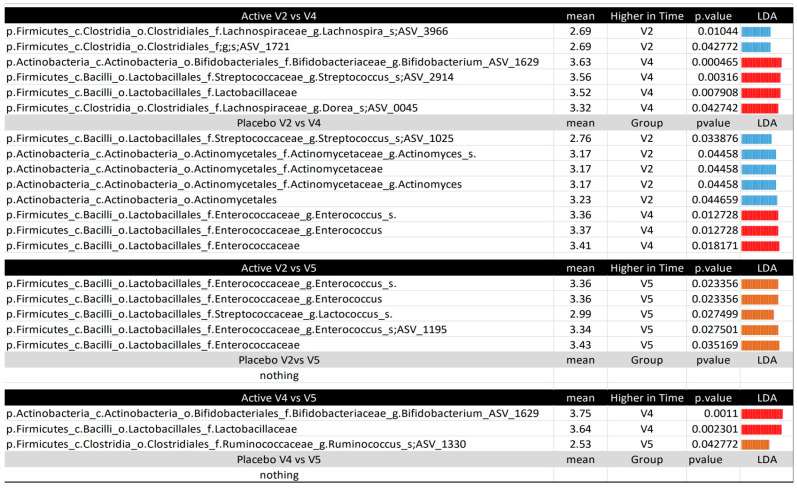
Microbiota comparison between the time points per treatment, according to the LEfSE. The figure displays the LEfSE results, with the mean values in the group with higher levels and the LDA for all comparisons between time points (V2, V4, V5) for each treatment (active or placebo). The taxonomy for all variables is provided in the following format: p, phylum; c, class; o, order; f, family; g, genus; s, species; ASV, amplicon sequence variant. The p__Actinobacteria.c__Actinobacteria.o__Bifidobacteriales.f__Bifidobacteriaceae.g__Bifidobacterium.ASV_1629 and the p__Firmicutes.c__Bacilli.o__Lactobacillales.f__Streptococcaceae.g__Streptococcus.s__.ASV_2914 were manually checked using BLAST v1.7.2, matching Bifidobacterium animalis subsp. lactis and Streptococcus thermophilus, respectively.

Using LEfSE, we confirmed the approximate overlapping results for the active treatment. However, in the placebo group, we observed an increase in *Enterococcus* and a decrease in *Actinomyces* (Figure 6). Regarding SCFAs, lactate increased in both treatments, while succinate increased after the active intake and acetate increased after the placebo intake. Comparing the results from the subsequent V4 and V5 time points, we noted no significant changes in the placebo treatment group. However, there was a decrease in *Bifidobacterium animalis* subsp. *lactis* and the *Lactobacillaceae* family, along with an increase in a species from the genus *Ruminococcus*, after the active treatment.

#### 3.5.1. Gut Microbiota Comparison Between Subjects After Treatment

**Figure 7:**
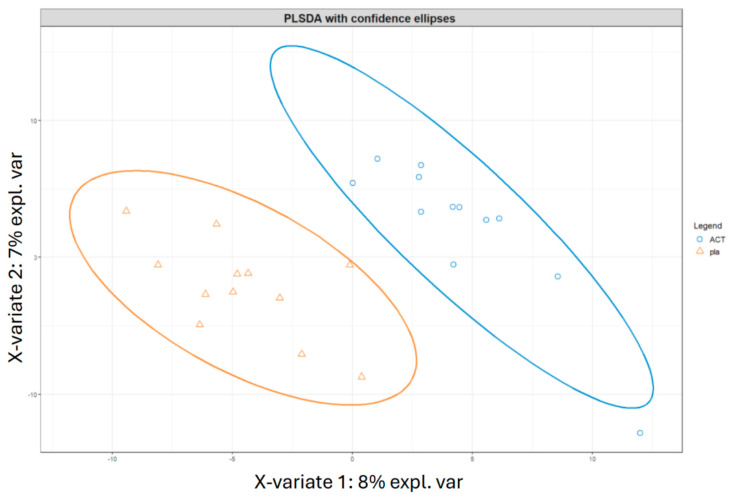
Classification of subjects at V4 from different treatments according to PLSDA analyses. The figure displays the clustering results from partial least squares discriminant analysis (PLSDA) of subjects at time point V4, comparing the two treatments, i.e., active and placebo. The PLSDA was conducted using an equal number of subjects from both treatment groups, with a total of 24 subjects. Subjects who received the active treatment are represented by blue circles, while those who received the placebo treatment are represented by orange triangles. ACT, active group; pla, placebo group.

The subjects at time point V4, week 12, were compared in an unpaired manner using the Mann–Whitney test to observe differences resulting from the different treatments (Supplementary Figure S3). The active group showed a significant increase in Actinobacteria and a decrease in Proteobacteria. To further explore these results, sparse partial least squares regression for discrimination analysis (sPLS-DA) was performed to determine whether the subjects could be clustered based on the treatment received and whether machine learning techniques could predict the treatment a subject underwent (Figure 7). Despite the small sample size, further reduced because of inadequate or missing samples, limiting the accuracy of the machine learning techniques, the ROC curve and an accuracy of 85% suggest the potential for classifying subjects based on the treatment. The most important variables for distinguishing between the two groups were *Bifidobacterium animalis* subsp. *lactis*, the alpha diversity index Faith_PD (which is based on the richness and phylogenetic relationships of the bacteria), and the SCFAs, particularly acetate and butyrate.

**Figure 8:**
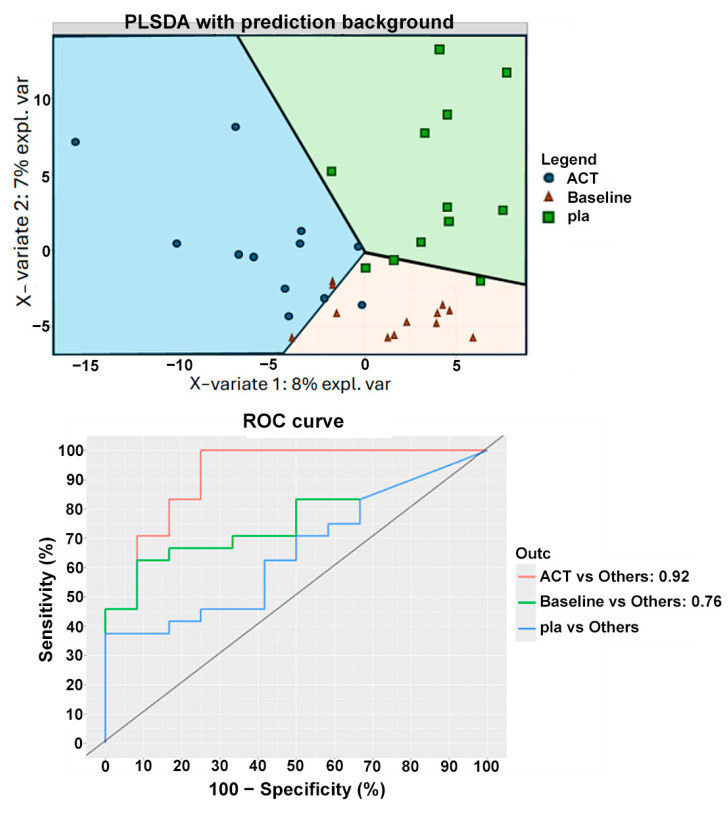
Classification of subjects at V4 from different treatments and baseline according to PLSDA analyses. The figure displays the clustering results from partial least squares discriminant analysis (PLSDA) of patients from the two groups at time point V4 and V2, with the least amount of clustering labeled as “baseline”. The PLSDA was conducted using an equal number of subjects from both treatment groups, with a total of 36 subjects. Subjects who received the active treatment are represented by blue points, while those who received the placebo treatment are represented by green squares, and the subjects at baseline are labeled with orange triangles. The figure also shows the ROC curve. ACT, active group; pla, placebo.

The sPLS-DA analysis was also performed on two groups: the V4 group after active treatment and the V4 group after placebo treatment, along with a third group from time point V2, which included all subjects before any treatment (Figure 8). The classification suggested that subjects in the V4 placebo group were more similar to subjects at V2 than to those in the V4 active treatment group. These results were confirmed by another machine learning algorithm, random forest, which showed an accuracy of 0.69 in distinguishing between the V2 group and the V4 placebo group. In contrast, the comparison between the V4 active and V4 placebo groups yielded a classification accuracy of 0.8, with *Bifidobacterium animalis* subsp. *lactis*, *Streptococcus thermophilus*, *Lachnospira*, Lactobacillaceae, and another undefined *Bifidobacterium* species emerging as the most important discriminatory variables. These findings are consistent with the results of the Mann–Whitney test used to compare the two groups.

#### 3.5.2. Correlation Between Microbial Potential Biomarkers After Active Treatment and Clinical Parameters, SCFAs, and Alpha-Diversity Indexes

The correlation analysis highlights the complex interactions between the gut microbiome, clinical parameters, SCFAs, and alpha-diversity indexes, emphasizing the potential of GM modulation in IBS treatment (Supplementary Figure S4). Starting with the bacteria administered with the active compound, *Bifidobacterium animalis* subsp. *lactis* was found to negatively correlate with butyrate, while *Streptococcus thermophilus* showed a negative correlation with citrulline.

Regarding the bacteria modulated by the active compound, a species within the Lachnospiraceae family was positively correlated with the physiological functioning of the subjects. *Lachnospira*, which decreased after the treatment, was positively correlated with zonulin and negatively correlated with the alpha diversity index of evenness. Additionally, *Ruminococcus* was positively correlated with the SF-36 domain regarding the pain index, where low levels indicate high abdominal pain, and high levels indicate no pain. Furthermore, an unidentified species within the *Dorea* genus increased after the treatment and was positively correlated with the alpha diversity index of evenness.

#### 3.5.3. PICRUSt-Based Prediction of Functional Pathways

KO pathways (*p*-value < 0.05), overexpressed in the active compared to the placebo group, were highlighted at the V2, V4, and V5 time points. In particular, at V2, the following KO pathways were identified: biosynthesis of unsaturated fatty acids; glycolysis/gluconeogenesis (K01792); messenger RNA biogenesis; nicotinate and nicotinamide metabolism; peptidases and inhibitors; propanoate metabolism (K123788); and the two-component system. At V4, the identified KOs included the following: arabinogalactan biosynthesis—mycobacterium (K02851); biosynthesis of unsaturated fatty acids; cysteine and methionine metabolism (K08969); messenger RNA biogenesis (K08300); peptidoglycan biosynthesis (K00887-K05363); phenylalanine, tyrosine and tryptophan biosynthesis (K04092); terpenoid backbone biosynthesis (K13787); and thiamine metabolism (K14153). Finally, at V5, the identified KOs were as follows: aminoacyl-tRNA biosynthesis (K02433-K02434-K02435); arginine biosynthesis (K00620); bacterial motility proteins (K02650-K02662); glycerophospholipid metabolism (K01048); histidine metabolism (K01496-K01523-K01693); novobiocin biosynthesis (K04517); peptidoglycan biosynthesis (K05364-K07009); peptidoglycan biosynthesis and degradation proteins (K07284); ribosome biogenesis (K06442); starch and sucrose metabolism (K00702); and transcription factors (K03705).

## 4. Discussion

IBS is a common bowel disorder with an as yet unclear pathogenesis; nevertheless, consistent evidence suggests that dysregulated gut–brain axis, dysbiosis, and gut barrier impairment play a major role in causing abdominal pain and impaired gut motility. To date, the criteria for IBS diagnosis have been revised, focusing on the presence of pain as a key symptom and dismissing more non-specific symptoms, such as abdominal discomfort, to define a diagnosis of IBS. However, the diagnostic criteria are only based on symptoms and do not include any objective measures or biomarkers; even applying the most updated Rome IV criteria, there is the possibility to include in the same clinical diagnosis of IBS patients showing different gut barrier function and gut microbiota status, in which gut barrier impairment could play a different role in determining symptoms. Our cohort reflects this gap of knowledge; even though every patient included met the Rome IV criteria, only a specific subgroup met the criteria for increased intestinal permeability based on the LaMa ratio (55% of patients with increased LaMa ratio at baseline) and serum zonulin (38.7% of patients with zonulin > 48 ng/mL at baseline). Our data support that IBS is an “umbrella diagnosis”, with different pathogenetic mechanisms and eventually, different responses to therapies. This heterogeneity is an important factor to take into consideration in our study with its small sample size, as it could limit the external reproducibility of the results in other cohorts. Based on the role of gut barrier dysfunction in IBS, gut microbiota modulation has gained increased interest in recent years. Diet, prebiotics, and antibiotics are valid alternatives for modulation, but probiotics constitute one of the most intriguing factors, due to their long-term safety profile, high availability, and high appreciability in patients. However, evidence of the efficacy of probiotics in IBS is not convincing, probably reflecting the difficulty in identifying the correct IBS population to be treated with the highest probability of response. In our cohort, improvement in symptoms based on IBS-SSS and PROs did not differ significantly between the active and the placebo group, with a very high placebo response up to 60% in regards to PROs, which can be explained by the global endpoint of PROs, the small sample size, the parallel design, and the relatively short run time, all factors that have been reported as associated with higher placebo response [28]. Considering this placebo response, the small sample size of our cohort could deeply impact the possibility of finding significant differences between groups. Interestingly, the patients treated with probiotics showed a progressive trend toward improvement in the percentage of days characterized by abnormal bowel movements, becoming significant 4 weeks after treatment discontinuation, compared to the results for the placebo group, as well as a trend toward improved stool consistency, which was even greater 4 weeks after treatment discontinuation.

Adding to our understanding of this data and generally, improving our grasp of the probiotic mechanism of action, we found a corresponding progressive decrease in serum zonulin levels during the entire observation period in the active group, reaching statistical significance compared to baseline levels at week 12 and week 16, and compared to the placebo levels after 4 weeks of treatment discontinuation, indicating a progressive restoration of the tight junctions and the epithelial barrier, according to the results of previous literature identifying zonulin as a reliable measure of intestinal permeability during probiotic therapies [27]. On the other hand, no significant variation in citrulline and PV-1 levels were was found. The absence of variation in PV-1 reflects that the endothelial and epithelial barriers are independent entities, as previously reported in the literature studying celiac patients, who only showed high PV-1 levels in cases of liver damage and elevated serum transaminases [9]. Furthermore, these results suggest that probiotics primarily act on the integrity of the epithelial barrier, as demonstrated by the significant reduction in zonulin, without affecting the enterocyte mass, as suggested by constant values of citrulline after treatment.

Using the LaMa ratio as a marker of intestinal permeability, we found a tendency toward fluctuating results at different time points, suggesting that intestinal permeability is a variable measure, reactive to the environment, and that it could be unstable in IBS. However, combined probiotic and vitamin D supplementation showed a stabilizing effect on intestinal permeability, as the active group showed similar percentages of patients with increased and normal permeability compared to baseline numbers (50% of patients with normal permeability at week 0 vs. 56.3% at week 12), whereas in the placebo group, a higher percentage of patients showed increased permeability at week 12, even if the percentages were similar at baseline (40% of patients with normal permeability at baseline vs. 20% at week 12). However, the LaMa ratio and zonulin results are not completely consistent. Even though the LaMa test has been used in IBS patients, and the literature supports its increased value in particular IBS populations [29,30,31], we should consider that this test more accurately reflects small bowel intestinal, and not colonic, permeability. These two sugar probe molecules could be degraded by colonic bacteria enzymes and could not effectively reflect colonic permeation [3]. Instead, zonulin could also be altered in colonic disease, as it previously demonstrated higher levels in ulcerative colitis patients compared to those in healthy controls [5] and in colorectal cancer patients [4], and this could explain the apparent inconsistency between LaMa and zonulin results in our cohort. Furthermore, another limitation of this study is that we did not perform an additional LaMa test at 16 weeks to compare the LaMa results to those for zonulin.

Interestingly, in our cohort, OttaBac^®^ plus vitamin D seemed to take a long time to act and surprisingly, it continued to act even after treatment discontinuation. This apparently inconsistent data could be explained by the effect of probiotics on the host microbial species. We know that probiotics can colonize the gut during assumption and that they rapidly disappear after treatment discontinuation, without a major impact on the host microbiome composition [32]. According to the existing literature, in our cohort, alpha diversity analysis, which measures intra-sample diversity, revealed no significant differences between time points in either treatment group. Similarly, beta diversity analysis using UniFrac did not show any clustering of subjects, indicating stable microbial diversity both within and between groups over time. This stability is consistent with the data in the existing literature, which suggests that short-term dietary interventions could not significantly alter the core microbiome in adults [33]. Despite the stability in diversity metrics, we observed the modulation of specific bacterial taxa between the active and placebo groups. In the active group, as expected, there was an increase in *Bifidobacterium animalis* subsp. *lactis* and *Streptococcus thermophilus*, both of which were included in the probiotic formulation. Notably, *Bifidobacterium* was present only during the treatment period, indicating that it does not persist in the subject’s microbiome after treatment ends. In contrast, *Streptococcus* was still detectable, suggesting that *Streptococcus* exhibits greater persistence than does *Bifidobacterium*, even though it is less common to find *Streptococcus* in such a persistent state [34]. However, less is known about the long-term effects on host gut microbiota after treatment discontinuation. In our cohort, several species within the Lachnospiraceae family increased post-treatment, except for the genus *Lachnospira*, which decreased. The Lachnospiraceae family plays a crucial role in SCFAs production. The literature indicates that elderly individuals with high levels of inflammation often experience depletion of this family, which can be restored with anti-inflammatory treatments such as a polyphenol-rich diet [22]. Furthermore, *Lachnospira* has been found to correlate with decreased zonulin parameters after active treatment. Interestingly, the genus *Dorea* exhibited a species-specific response: one species increased in the probiotic group, while the genus overall increased in the placebo group. The role of *Dorea* in the gut is controversial, as it has been associated with increased gas production and intestinal permeability, factors thought to contribute to IBS pathophysiology. *Dorea* is involved in the fermentation of complex carbohydrates, which can lead to gas and SCFAs production [35,36]. Excessive SCFAs can have both positive and negative effects on gut health in non-constipated IBS patients, as described by Gargari et al. [37,38]. Notably, we found a positive correlation between *Dorea* and the alpha-diversity evenness index. Healthy guts typically exhibit high alpha diversity, associated with improved resilience and functionality, and better health outcomes [39]. Although Ruminococcaceae, known for fiber degradation and SCFA production, also increased in the active group, we did not observe a corresponding increase in SCFA production. We noted a positive correlation between this family and the pain index, suggesting a potential role in pain relief. Lactic acid levels increased in both groups, while acetate levels rose only in the placebo group. LEfSE analysis confirmed the modulation patterns observed, indicating minimal changes in SCFA levels between the placebo and active groups, but still showing bacterial modulation in the active group.

Regarding the differences observed post-treatment between the groups, the machine learning method confirmed a deeper change in microbiota composition after conclusion of treatment in the active group compared to that in the placebo group. Specifically, Proteobacteria decreased, and Actinobacteria increased in the active group. Actinobacteria, particularly the *Bifidobacterium* genus, are often found to be decreased in IBS patients [40], while Proteobacteria, which includes Gram-negative bacteria, are considered potentially harmful to the intestine [41]. Correlation analysis revealed complex interactions between the gut microbiome, clinical parameters, SCFAs, and alpha diversity indexes, suggesting that microbiome modulation may have the potential to improve the symptoms of non-constipated IBS. As overall microbial diversity remained stable, specific bacteria were significantly influenced by probiotic and vitamin D3 supplementation. Further long-term studies are needed to explore the sustained effects and clinical implications of these treatments. PICRUST prediction highlighted a switch regarding the SCFA producers’ metabolism at V5 with reference to “starch and sucrose metabolism” KO, suggesting an association with Ruminococcaceae, Erysipelotricaceae, and other SCFA-producing bacteria [42]. Moreover, novobiocin biosynthesis KO was also detected only at V5, suggesting the biosynthesis of antimicrobial peptides after the end of the treatment, with a shift in the ecological and functional framework between treatment discontinuation (V4) and the 4-week follow-up (V5) time points.

## 5. Conclusions

In our cohort, we found that first, probiotics can colonize the gut, as we found probiotic-related species after only 12 weeks of therapy. At the early stages, the probiotic species has probably not started to significantly impact the host microbiota and gut barrier. These interactions require time to develop and could become more and more significant with time, even after the interruption of probiotic oral supplementation, as at 16 weeks, after a 4-week washout, probiotic species started to disappear, but a different microbial composition could be retrieved compared to the results obtained at baseline. Consistently, the functional change in gut microbiota seemed to be delayed compared to the ecologic change, as demonstrated by the overexpression of the KO pathways of the biosynthesis of novobiocin and the ABC transporter at week 16 in the active group compared to the results for week 12. Possibly, it is at week 16 that the gut microbiota changes begin to positively and significantly impact barrier function, as demonstrated by further improvement in gut barrier function, based on zonulin levels, at week 16. At the same time, the supplementation of vitamin D could simultaneously act on deeper layers of the gut barrier through the activation of the vitamin D receptors on the epithelial and immune cells. In fact, vitamin D demonstrated a pleiotropic effect on the regulation of the integrity of the intestinal epithelial cells, the induction of immunotolerance and, generally, on the regulation of the immune system, with a consequent global improvement in gut barrier function [43] and permeability. Vitamin D deficiency is common in patients suffering from IBS, with an inverse correlation between serum vitamin D and IBS symptom severity [44]. Thus, the supplementation of vitamin D in IBS-D has a strong rationale, even if existing clinical trials have provided conflicting results regarding its impact on IBS symptoms, probably due to heterogeneity and relatively small sample sizes of included patients [45,46]. Unfortunately, our study only includes patients treated with a combination treatment comprising vitamin D and probiotics; consequently, we cannot discriminate which effects could be related to vitamin D and which to probiotics.

In this scenario, it is not surprising that clinical improvement followed the timeline of gut barrier improvement. It is likely that the beneficial effect on the gut barrier temporarily precedes clinical effects, and we can speculate that a hypothetically longer follow-up, beyond week 16, would have provided the opportunity to detect a deeper clinical effect related to an improved gut barrier. Unfortunately, our time of observation stopped at this time point, and we are not able to evaluate whether patients would have experienced a clinical improvement in subsequent weeks.

## Figures and Tables

**Table 1:** Demographic characteristics of the intent-to-treat population.

		Summary Statistics	Total (*n* = 31)	Placebo (*n* = 15)	Active (*n* = 16)
Sex	F	%, *n*	54.8% (17/31)	53.3% (8/15)	56.3% (9/16)
	M	%, *n*	45.2% (14/31)	46.7% (7/15)	43.8% (7/16)
Age		Mean (SD)	36.26 (10.652)	37.40 (10.439)	35.19 (11.077)
Weight (kg)		Mean (SD)	68.14 (11.074)	68.26 (10.676)	68.03 (11.784)
Height (cm)		Mean (SD)	169.73 (7.625)	167.64 (5.852)	171.56 (8.664)
Rome IV	IBS-D	%, *n*	71.0 (22/31)	73.3 (11/15)	68.8 (11/16)
	IBS-M	%, *n*	29.0 (9/31)	26.7 (4/15)	31.3 (5/16)
Disease duration (months)		Mean (SD)	538.19 (649.237)	592.27 (681.304)	487.50 (635.706)
IBS-SSS		Mean (SD)	243.40 (88.10)	250.08 (78.42)	237.17 (98.63)
Mild (<174)		%, *n*	29.03 (9/31)	9.68 (3/31)	19.35 (6/31)
Moderate (175–300)		%, *n*	32.26 (10/31)	19.35 (6/31)	12.90 (4/31)
Severe (>300)		%, *n*	32.26 (10/31)	16.13 (5/31)	16.13 (5/31)
Missing		%, *n*	6.45 (2/31)	3.23 (1/31)	3.23 (1/31)
Stool consistency (Bristol stool scale)		Mean (SD)	4.48 (1.25)	4.14 (1.44)	4.80 (1.01)
1–2		%, *n*	1	1	0
3–4–5		%, *n*	18	10	8
6–7		%, *n*	8	2	6
Missing		%, *n*	4	2	2

IBS-SSS, irritable bowel syndrome severity scoring system; *n*, number; SD, standard deviation.

**Table 2:** Marginal mean of SF-36 domains by week and variation from baseline by group (ITT).

		Placebo				Active				Active vs. Placebo	
				Change from Baseline				Change from Baseline			
Parameter	Visit	*n*	Mean (se)	Mean (se)	*p*-Value	*n*	Mean(se)	Mean (se)	*p*-Value	Mean Diff of Variation Active-Placebo (95% CI)	*p*-Value
Physical functioning	0	15	92.98 (1.59)			15	93.02 (1.59)				
	12	15	91.65 (1.59)	−1.33 (2.06)	0.5210	16	94.35 (1.59)	1.33 (2.06)	0.5210	2.67(−3.18; 8.52)	0.3649
	16	15	90.98 (1.59)	−2.00 (2.06)	0.3368	16	96.02 (1.59)	3.00 (2.06)	0.1517	5(−0.85; 10.85)	0.0923
Role—physical	0	15	58.44 (7.88)			15	54.90 (7.88)				
	12	15	61.77 (7.88)	3.33 (10.31)	0.7477	16	81.56 (7.88)	26.67 (10.31)	0.0123	23.33(−5.88; 52.54)	0.1152
	16	15	65.10 (7.88)	6.67 (10.31)	0.5205	16	88.23 (7.88)	33.33 (10.31)	0.0021	26.67(−2.54; 55.88)	0.0727
Pain index	0	15	54.73 (4.42)			15	54.68 (4.42)				
	12	15	63.67 (4.42)	8.93 (6.05)	0.1452	16	62.39 (4.57)	7.71 (6.16)	0.2160	−1.22(−18.52; 16.07)	0.8878
	16	15	59.60 (4.42)	4.87 (6.05)	0.4243	16	64.48 (4.42)	9.80 (6.05)	0.1109	4.93(−12.21; 22.06)	0.5666
General health perceptions	0	15	57.99 (2.94)			15	58.87 (2.94)				
	12	15	60.79 (3.04)	2.80 (3.90)		16	64.33 (2.94)	5.47 (3.82)	0.1581	2.67(−8.27; 13.61)	0.6268
	16	15	62.69 (3.15)	4.70 (3.98)		16	64.00 (2.94)	5.13 (3.82)	0.1846	0.44(−10.63; 11.5)	0.9371
Vitality	0	15	48.35 (3.16)			15	49.98 (3.16)				
	12	15	54.02 (3.16)	5.67 (4.14)	0.1766	16	54.98 (3.16)	5.00 (4.14)	0.2323	−0.67(−12.4; 11.06)	0.9098
	16	15	57.35 (3.16)	9.00 (4.14)	0.0340	16	56.98 (3.16)	7.00 (4.14)	0.0965	−2(−13.73; 9.73)	0.7340
Social functioning	0	15	60.39 (4.65)			15	57.11 (4.65)				
	12	15	64.55 (4.65)	4.17 (5.99)	0.4898	16	69.61 (4.65)	12.50 (5.99)	0.0416	8.33(−8.65; 25.31)	0.3298
	16	15	61.22 (4.65)	0.83 (5.99)	0.8899	16	77.11 (4.65)	20.00 (5.99)	0.0015	19.17(2.19; 36.15)	0.0277
Role—emotional	0	15	43.06 (7.11)			15	52.49 (7.11)				
	12	15	76.40 (7.11)	33.33 (9.97)	0.0015	16	68.05 (7.11)	15.56 (9.97)	0.1244	−17.78(−46.03; 10.47)	0.2127
	16	15	69.73 (7.11)	26.67 (9.97)	0.0098	16	76.94 (7.11)	24.44 (9.97)	0.0174	−2.22(−30.47; 26.03)	0.8754
Mental health index	0	15	61.01 (2.65)			15	61.99 (2.65)				
	12	15	64.61 (2.65)	3.60 (3.54)	0.3137	16	67.46 (2.65)	5.47 (3.54)	0.1282	1.87(−8.16; 11.9)	0.7107
	16	15	62.74 (2.65)	1.73 (3.54)	0.6264	16	70.33 (2.65)	8.33 (3.54)	0.0221	6.6(−3.43; 16.63)	0.1929

CI, confidence interval; *n*, number; se, standard error.

## Data Availability

The data presented in this study are available on request from the corresponding author.

## Supplementary Materials

The following supporting information can be downloaded at: https://www.mdpi.com/article/10.3390/nu17101708/s1, Supplementary Table S1: Distribution of patients according to Bristol stool chart before and after therapy; Supplementary Table S2: Marginal mean of abdominal pain, bloating, and Irritable Bowel Syndrome Symptom Severity Score (IBS-SSS) at each week and variation from baseline by group, intent-to-treat; Supplementary Table S3: Marginal mean of abdominal pain, bloating, and Irritable Bowel Syndrome Symptom Severity Score (IBS-SSS) at each week and variation from baseline by group, per-protocol; Supplementary Figure S1: Microbiota comparison between the time points per treatment by Wilcoxon test. The figure displays the Wilcoxon results with the median values for all comparisons between time points (V2, V4, V5) for each treatment (Active or Placebo). The medians are labeled in blue or red, indicating high or low bacterial abundance, respectively. The taxonomy for all variables is provided in the following format: p, phylum; c, class; o, order; f, family; g, genus; s, species; ASV, amplicon sequence variant. The p__Actinobacteria.c__Actinobacteria.o__Bifidobacteriales.f__Bifidobacteriaceae.g__Bifidobacterium.ASV_ 1629 and the p__Firmicutes.c__Bacilli.o__Lactobacillales.f__Streptococcaceae.g__Streptococcus.s__.ASV_2914 were manually checked using BLAST, matching Bifidobacterium animalis subsp. lactis and Streptococcus thermophilus, respectively; Supplementary Figure S2: SCFAs comparison between the time points per treatment by Wilcoxon or Ttest. The figure displays the Wilcoxon or Ttest (in dependence on the Shapiro francia test of normality of the data) results with the median values for all comparisons between time points (V2, V4, V5) for each treatment (Active or Placebo). The medians or means (in dependence on the Shapiro francia test of normality of the data) are labeled in blue or red, indicating high or low SCFAs abundance, respectively. The SCFAs under analyses are: Lac, lactic acid; AA, acetic acid; SA, succinic acid, PA, propionic acid; BA, butyric acid; iBA iso-butyric acid; iVA, iso-valeric acid; VA, Valeric acid. In bold the significance with P < 0.05 and in blue the results from the Ttest; Supplementary Figure S3: Microbiota comparison between the treatment on time V4 by Mann-Whitney test. The figure displays the Mann-Whitney results with the median values for all comparisons between treatments (Active, Placebo) at time V4. The medians are labeled in blue or red, indicating high or low bacterial abundance, respectively. The taxonomy for all variables is provided in the following format: p, phylum; c, class; o, order; f, family; g, genus; s, species; ASV, amplicon sequence variant. The p__Actinobacteria.c__Actinobacteria.o__Bifidobacteriales.f__Bifidobacteriaceae.g__Bifidobacterium. ASV_ 1629 was manually checked using BLAST, matching Bifidobacterium animalis subsp. lactis. The number of subjects took in consideration for the analyses was 26 (14 Placebo, 12 Active); Supplementary Figure S4: Kendall correlation at baseline. This figure presents the correlations determined by the Kendall test between clinical variables, SCFAs, and alpha diversity indexes with bacteria that were found to differ significantly between time points V2 and V4 in the two treatments, as determined by the Wilcoxon test. The median levels of specific bacteria at V2 and V4 are indicated by red and blue, respectively, with red representing a higher level and blue representing a lower level. In the correlation matrix, red and green colors indicate negative and positive correlations, respectively. The numbers represent the *p*-values, where a negative correlation is indicated by a *p*-value < 0. The table includes only those bacteria that have at least one significant correlation with the listed variables.
