# Supplementary material for: Multistrain Probiotics Plus Vitamin D Improve Gut Barrier Function and Gut Microbiota Composition in Irritable Bowel Syndrome Without Constipation: Results from a Double-Blind, Randomized, Placebo-Controlled Trial
Source: Nutrients. 2025 May 18;17(10):1708. doi: 10.3390/nu17101708 (PMC12114473; doi:10.3390/nu17101708)
Supplement: Supplementary file 1 [file nutrients-17-01708-s001.zip › Ottabac_Supplementary Table S1.pdf]

Supplementary Table S1. Distribution of patients according to Bristol stool chart before and after therapy.

ITT							PP						
		**Placebo**		**Active**					**Placebo**		**Active**		
	**Stool**	**n**	**%**	**n**	**%**	**pvalue**		**Stool**	**n**	**%**	**n**	**%**	**pvalue**
week 0							week 0						
	1-2	1	7,7	0	0,0	0,203		1-2	1	7,7	0	0,0	0,249
	3-4-5	10	76,9	8	57,1			3-4-5	10	76,9	7	58,3	
	6-7	2	15,4	6	42,9			6-7	2	15,4	5	41,7	
week 12							week 12						
	1-2	1	7,7	0	0,0	0,0935		1-2	1	7,7	0	0,0	0,218
	3-4-5	12	92,3	12	75,0			3-4-5	12	92,3	11	84,6	
	6-7	0	0,0	4	25,0			6-7	0	0,0	2	15,4	
week 16							week 16						
	1-2	1	9,1	0	0,0	0,152		1-2	1	9,1	0	0,0	0,176
	3-4-5	8	72,7	12	100,0			3-4-5	8	72,7	11	100,0	
	6-7	2	18,2	0	0,0			6-7	2	18,2	0	0,0	

ITT, intention-to-treat; PP, per-protocol.
