# Supplementary material for: Multistrain Probiotics Plus Vitamin D Improve Gut Barrier Function and Gut Microbiota Composition in Irritable Bowel Syndrome Without Constipation: Results from a Double-Blind, Randomized, Placebo-Controlled Trial
Source: Nutrients. 2025 May 18;17(10):1708. doi: 10.3390/nu17101708 (PMC12114473; doi:10.3390/nu17101708)
Supplement: Supplementary file 1 [file nutrients-17-01708-s001.zip › Ottabac_Supplementary Table S2.pdf]

Supplementary Table S2. Marginal mean of abdominal pain, bloating, and Irritable Bowel Syndrome Symptom Severity Score (IBS-SSS) at each week and variation from baseline by group, intent-to-treat.

		Placebo				Active					
				change from baseline				change from baseline		Active vs Placebo	
**Parameter**	**Week**	**n**	**mean (se)**	**mean (se)**	**pvalue**	**n**	**mean(se)**	**mean (se)**	**pvalue**	**Mean Diff of variation Active - Placebo (95% CI)**	**pvalue**
Abdominal	0	14	39.14 (7.77)			15	41.78 (7.66)				
pain	1	13	32.54 (8.01)	-6.60 (8.41)	0.4332	15	32.53 (7.51)	-9.25 (8.05)	0.2514	-2.65 (-25.54; 20.24)	0.8199
intensity	2	13	32.54 (8.01)	-6.60 (8.41)	0.4332	15	40.93 (7.51)	-0.85 (8.05)	0.9161	5.75 (-17.14; 28.64)	0.6218
	3	13	28.16 (8.01)	-11.0 (8.41)	0.1923	15	29.67 (7.51)	-12.1 (8.05)	0.1332	-1.13 (-24.03; 21.76)	0.9224
	4	13	29.01 (8.01)	-10.1 (8.41)	0.2288	15	23.80 (7.51)	-18.0 (8.05)	0.0261	-7.85 (-30.74; 15.04)	0.5007
	5	13	43.61 (8.43)	4.47 (8.81)	0.6124	15	30.77 (7.68)	-11.0 (8.21)	0.1807	-15.48 (-39.17; 8.2)	0.1995
	6	13	29.77 (8.01)	-9.37 (8.41)	0.2656	15	28.85 (7.68)	-12.9 (8.21)	0.1162	-3.56 (-26.67; 19.55)	0.7621
	7	13	33.54 (8.01)	-5.60 (8.41)	0.5055	15	36.70 (7.68)	-5.08 (8.21)	0.5370	0.53 (-22.58; 23.64)	0.9642
	8	13	24.08 (8.01)	-15.1 (8.41)	0.0740	15	31.49 (7.68)	-10.3 (8.21)	0.2111	4.77 (-18.34; 27.89)	0.6848
	9	13	36.33 (8.20)	-2.81 (8.59)	0.7441	15	34.42 (7.68)	-7.36 (8.21)	0.3706	-4.56 (-27.93; 18.82)	0.7018
	10	13	35.78 (8.01)	-3.36 (8.41)	0.6895	15	37.16 (7.85)	-4.62 (8.38)	0.5815	-1.26 (-24.61; 22.08)	0.9153
	11	13	35.46 (8.01)	-3.68 (8.41)	0.6618	14	34.03 (7.68)	-7.75 (8.21)	0.3458	-4.07 (-27.19; 19.04)	0.7291
	12	13	34.96 (8.01)	-4.18 (8.41)	0.6193	14	35.95 (7.68)	-5.83 (8.21)	0.4786	-1.65 (-24.76; 21.47)	0.8887
	13	12	36.71 (8.21)	-2.43 (8.60)	0.7773	12	41.52 (8.06)	-0.26 (8.57)	0.9757	2.17 (-21.7; 26.04)	0.8582
	14	10	28.86 (8.70)	-10.3 (9.07)	0.2576	12	34.54 (8.29)	-7.24 (8.79)	0.4109	3.04 (-21.79; 27.87)	0.8098
	15	8	32.61 (9.38)	-6.53 (9.73)	0.5027	10	28.95 (8.56)	-12.8 (9.05)	0.1570	-6.31 (-32.43; 19.82)	0.6353
	16	6	28.34 (10.43)	-10.8 (10.74)	0.3150	9	28.43 (8.87)	-13.4 (9.33)	0.1530	-2.55 (-30.52; 25.41)	0.8577
Abdominal	0	14	37.15 (8.53)			15	39.33 (8.24)				
pain	1	13	28.96 (8.75)	-8.19 (8.06)	0.3104	15	34.67 (8.24)	-4.66 (7.55)	0.5374	3.52 (-18.19; 25.24)	0.7499
frequency	2	13	28.96 (8.75)	-8.19 (8.06)	0.3104	15	43.33 (8.24)	4.00 (7.55)	0.5962	12.19 (-9.53; 33.91)	0.2704

	3	13	33.58 (8.75)	-3.57 (8.06)	0.6580	15	34.67 (8.24)	-4.66 (7.55)	0.5374	-1.09 (-22.81; 20.62)	0.9213
	4	13	29.73 (8.75)	-7.42 (8.06)	0.3580	15	24.00 (8.24)	-15.3 (7.55)	0.0431	-7.91 (-29.63; 13.8)	0.4742
	5	13	40.58 (8.92)	3.43 (8.24)	0.6776	15	29.33 (8.24)	-9.99 (7.55)	0.1865	-13.42 (-35.4; 8.55)	0.2305
	6	13	27.42 (8.75)	-9.72 (8.06)	0.2283	15	26.00 (8.24)	-13.3 (7.55)	0.0784	-3.6 (-25.32; 18.11)	0.7443
	7	13	32.04 (8.75)	-5.11 (8.06)	0.5265	15	31.33 (8.24)	-8.00 (7.55)	0.2904	-2.89 (-24.6; 18.83)	0.7940
	8	13	25.11 (8.75)	-12.0 (8.06)	0.1362	15	37.75 (8.38)	-1.58 (7.70)	0.8380	10.46 (-11.46; 32.37)	0.3487
	9	13	32.04 (8.75)	-5.11 (8.06)	0.5264	15	36.32 (8.38)	-3.00 (7.70)	0.6966	2.11 (-19.81; 24.02)	0.8502
	10	13	34.35 (8.75)	-2.80 (8.06)	0.7283	15	34.67 (8.24)	-4.66 (7.55)	0.5374	-1.86 (-23.58; 19.85)	0.8663
	11	13	32.04 (8.75)	-5.11 (8.06)	0.5264	14	34.89 (8.38)	-4.43 (7.70)	0.5652	0.68 (-21.24; 22.59)	0.9515
	12	13	30.50 (8.75)	-6.65 (8.06)	0.4098	14	31.32 (8.38)	-8.00 (7.70)	0.2992	-1.36 (-23.27; 20.56)	0.9033
	13	12	32.46 (8.92)	-4.69 (8.24)	0.5694	12	36.96 (8.70)	-2.37 (8.05)	0.7690	2.33 (-20.32; 24.97)	0.8400
	14	10	26.90 (9.33)	-10.2 (8.69)	0.2391	12	40.28 (8.70)	0.95 (8.05)	0.9059	11.2 (-12.09; 34.48)	0.3450
	15	8	31.51 (9.92)	-5.63 (9.32)	0.5458	10	28.28 (9.12)	-11.0 (8.50)	0.1947	-5.41 (-30.22; 19.39)	0.6680
	16	6	23.32 (10.83)	-13.8 (10.28)	0.1795	9	26.92 (9.39)	-12.4 (8.80)	0.1593	1.42 (-25.18; 28.03)	0.9163
Abdominal distension	0	14	44.57 (7.66)			15	42.08 (7.67)				
	1	13	39.85 (8.03)	-4.72 (7.66)	0.5380	15	44.68 (7.53)	2.60 (7.42)	0.7267	7 (-13.66; 28.29)	0.4930
	2	13	42.60 (8.03)	-1.97 (7.66)	0.7970	15	38.60 (7.40)	-3.49 (7.31)	0.6338	-2 (-22.34; 19.31)	0.8864
	3	13	33.42 (7.87)	-11.1 (7.49)	0.1377	15	34.93 (7.40)	-7.15 (7.31)	0.3287	4 (-16.59; 24.58)	0.7028
	4	13	30.73 (7.87)	-13.8 (7.49)	0.0655	15	33.53 (7.40)	-8.55 (7.31)	0.2429	5 (-15.29; 25.87)	0.6137
	5	13	41.77 (8.21)	-2.80 (7.85)	0.7217	15	26.60 (7.40)	-15.5 (7.31)	0.0348	-13 (-33.78; 8.41)	0.2377
	6	13	32.16 (8.03)	-12.4 (7.66)	0.1060	15	35.88 (7.54)	-6.20 (7.45)	0.4061	6 (-14.8; 27.22)	0.5614
	7	13	33.66 (7.87)	-10.9 (7.49)	0.1460	15	36.24 (7.54)	-5.84 (7.45)	0.4337	5 (-15.71; 25.85)	0.6314
	8	13	23.04 (7.87)	-21.5 (7.49)	0.0043	15	34.96 (7.54)	-7.13 (7.45)	0.3396	14 (-6.38; 35.18)	0.1737
	9	13	37.18 (8.03)	-7.39 (7.66)	0.3356	15	46.99 (7.68)	4.91 (7.58)	0.5179	12 (-8.9; 33.49)	0.2547
	10	13	39.42 (7.87)	-5.15 (7.49)	0.4926	15	40.54 (7.84)	-1.54 (7.77)	0.8430	4 (-17.62; 24.83)	0.7385
	11	13	40.12 (7.87)	-4.45 (7.49)	0.5526	14	39.53 (7.54)	-2.56 (7.45)	0.7318	2 (-18.88; 22.68)	0.8576
	12	13	34.96 (7.87)	-9.61 (7.49)	0.2006	14	43.10 (7.54)	1.02 (7.45)	0.8917	11 (-10.16; 31.4)	0.3154
	13	12	35.46 (8.03)	-9.11 (7.66)	0.2350	12	45.29 (7.84)	3.20 (7.77)	0.6806	12 (-9.14; 33.77)	0.2598

	14	10	29.26 (8.43)	-15.3 (8.08)	0.0591	12	56.29 (7.84)	14.20 (7.77)	0.0685	30 (7.46; 51.56)	0.0088
	15	8	23.05 (9.00)	-21.5 (8.67)	0.0135	10	39.35 (8.25)	-2.73 (8.19)	0.7389	19 (-4.67; 42.24)	0.1162
	16	6	30.94 (9.87)	-13.6 (9.57)	0.1552	9	26.06 (8.51)	-16.0 (8.44)	0.0584	-2 (-27.48; 22.69)	0.8514
Satisfaction of bowel habits	0	14	63.71 (5.03)			15	69.30 (4.94)				
	1	13	59.74 (5.16)	-3.98 (4.82)	0.4094	15	65.07 (4.86)	-4.24 (4.60)	0.3583	-0.26 (-13.36; 12.85)	0.9693
	2	13	58.89 (5.16)	-4.82 (4.82)	0.3172	15	65.40 (4.86)	-3.90 (4.60)	0.3973	0.92 (-12.18; 14.03)	0.8899
	3	13	54.20 (5.16)	-9.52 (4.82)	0.0489	15	64.20 (4.86)	-5.10 (4.60)	0.2686	4.42 (-8.69; 17.52)	0.5080
	4	13	56.27 (5.16)	-7.44 (4.82)	0.1233	15	62.07 (4.86)	-7.24 (4.60)	0.1170	0.2 (-12.9; 13.31)	0.9755
	5	13	62.65 (5.26)	-1.06 (4.92)	0.8292	15	61.36 (5.03)	-7.94 (4.79)	0.0985	-6.88 (-20.39; 6.64)	0.3176
	6	13	53.81 (5.16)	-9.90 (4.82)	0.0405	15	61.09 (4.94)	-8.21 (4.70)	0.0814	1.69 (-11.54; 14.92)	0.8019
	7	13	58.58 (5.16)	-5.13 (4.82)	0.2874	15	63.26 (5.04)	-6.04 (4.80)	0.2085	-0.91 (-14.28; 12.46)	0.8935
	8	13	56.04 (5.16)	-7.67 (4.82)	0.1121	15	59.41 (4.94)	-9.89 (4.70)	0.0360	-2.22 (-15.45; 11.01)	0.7417
	9	13	61.85 (5.16)	-1.86 (4.82)	0.6991	15	59.23 (4.94)	-10.1 (4.70)	0.0328	-8.21 (-21.44; 5.03)	0.2235
	10	13	58.74 (5.16)	-4.98 (4.82)	0.3020	15	59.39 (5.14)	-9.91 (4.91)	0.0442	-4.93 (-18.45; 8.59)	0.4740
	11	13	57.29 (5.26)	-6.43 (4.92)	0.1926	14	62.34 (5.04)	-6.97 (4.80)	0.1472	-0.54 (-14.06; 12.98)	0.9375
	12	13	53.58 (5.16)	-10.1 (4.82)	0.0361	14	60.87 (5.04)	-8.43 (4.80)	0.0797	1.7 (-11.66; 15.07)	0.8022
	13	12	54.88 (5.26)	-8.84 (4.92)	0.0736	12	67.47 (5.14)	-1.83 (4.90)	0.7097	7.01 (-6.66; 20.68)	0.3140
	14	10	52.22 (5.67)	-11.5 (5.37)	0.0329	12	61.64 (5.14)	-7.66 (4.90)	0.1192	3.83 (-10.46; 18.13)	0.5982
	15	8	48.77 (6.11)	-14.9 (5.83)	0.0108	10	61.77 (5.40)	-7.53 (5.18)	0.1468	7.41 (-7.92; 22.74)	0.3425
	16	6	52.20 (6.83)	-11.5 (6.58)	0.0808	9	54.94 (5.76)	-14.4 (5.54)	0.0100	-2.84 (-19.76; 14.08)	0.7413
Interference on life	0	14	65.50 (5.34)			15	65.73 (5.23)				
	1	13	64.07 (5.46)	-1.42 (4.58)	0.7564	15	62.60 (5.16)	-3.13 (4.38)	0.4751	-1.71 (-14.18; 10.76)	0.7879
	2	13	58.53 (5.77)	-6.97 (4.94)	0.1597	15	66.17 (5.41)	0.44 (4.68)	0.9252	7.41 (-5.98; 20.8)	0.2771
	4	13	55.33 (5.65)	-10.2 (4.81)	0.0352	15	60.53 (5.16)	-5.20 (4.38)	0.2361	4.97 (-7.82; 17.76)	0.4449
	5	13	61.07 (5.65)	-4.43 (4.81)	0.3573	15	56.52 (5.32)	-9.21 (4.57)	0.0446	-4.78 (-17.82; 8.26)	0.4716
	6	13	58.77 (5.46)	-6.73 (4.58)	0.1430	15	63.08 (5.24)	-2.65 (4.48)	0.5538	4.08 (-8.53; 16.68)	0.5248
	7	13	52.77 (5.46)	-12.7 (4.58)	0.0058	15	58.69 (5.24)	-7.05 (4.48)	0.1164	5.69 (-6.92; 18.29)	0.3755
	8	13	57.57 (5.46)	-7.92 (4.58)	0.0849	15	62.44 (5.24)	-3.30 (4.48)	0.4621	4.63 (-7.98; 17.23)	0.4706

	9	13	59.19 (5.46)	-6.31 (4.58)	0.1698	15	56.90 (5.24)	-8.83 (4.48)	0.0493	-2.52 (-15.13; 10.08)	0.6941
	10	13	56.77 (5.46)	-8.73 (4.58)	0.0577	15	55.24 (5.32)	-10.5 (4.57)	0.0224	-1.76 (-14.49; 10.98)	0.7864
	11	13	59.38 (5.46)	-6.12 (4.58)	0.1831	14	57.04 (5.24)	-8.69 (4.48)	0.0531	-2.57 (-15.18; 10.03)	0.6884
	12	13	54.46 (5.55)	-11.0 (4.69)	0.0192	14	58.83 (5.32)	-6.90 (4.55)	0.1306	4.14 (-8.72; 17)	0.5273
	13	12	57.72 (5.55)	-7.78 (4.69)	0.0982	12	64.09 (5.41)	-1.64 (4.68)	0.7257	6.14 (-6.89; 19.16)	0.3548
	14	10	55.85 (5.91)	-9.64 (5.11)	0.0601	12	67.55 (5.41)	1.82 (4.68)	0.6979	11.46 (-2.17; 25.09)	0.0990
	15	8	59.87 (6.29)	-5.63 (5.55)	0.3116	10	57.32 (5.64)	-8.41 (4.92)	0.0881	-2.79 (-17.38; 11.81)	0.7075
	16	6	56.19 (6.93)	-9.31 (6.27)	0.1385	9	50.53 (5.95)	-15.2 (5.28)	0.0043	-5.9 (-22.02; 10.23)	0.4724
IBS-SSS	0	14	250.2 (29.08)			15	236.9 (28.10)				
	1	13	221.7 (29.82)	-28.5 (27.57)	0.3021	15	236.8 (28.10)	-0.09 (25.86)	0.9973	28.4 (-45.92; 102.72)	0.4529
	2	13	205.9 (29.82)	-44.3 (27.57)	0.1093	15	240.5 (28.10)	3.58 (25.86)	0.8900	47.83 (-26.48; 122.15)	0.2065
	3	13	149.4 (29.82)	-101 (27.57)	0.0003	15	163.6 (28.10)	-73.3 (25.86)	0.0048	27.43 (-46.89; 101.75)	0.4685
	4	13	190.7 (29.82)	-59.5 (27.57)	0.0316	15	204.1 (28.10)	-32.8 (25.86)	0.2051	26.67 (-47.65; 100.98)	0.4809
	5	13	241.9 (30.39)	-8.31 (28.18)	0.7683	15	187.8 (28.10)	-49.1 (25.86)	0.0583	-40.81 (-116.02; 34.4)	0.2867
	6	13	199.8 (29.82)	-50.4 (27.57)	0.0683	15	202.7 (28.10)	-34.3 (25.86)	0.1861	16.15 (-58.16; 90.47)	0.6693
	7	13	210.6 (29.82)	-39.6 (27.57)	0.1521	15	210.4 (28.10)	-26.6 (25.86)	0.3051	13.01 (-61.31; 87.33)	0.7309
	8	13	185.9 (29.82)	-64.2 (27.57)	0.0204	15	221.1 (28.56)	-15.8 (26.36)	0.5482	48.37 (-26.63; 123.37)	0.2056
	9	13	220.9 (29.82)	-29.3 (27.57)	0.2893	15	225.6 (28.56)	-11.3 (26.36)	0.6692	17.98 (-57.02; 92.98)	0.6376
	10	13	225.1 (29.82)	-25.1 (27.57)	0.3631	15	194.8 (28.10)	-42.1 (25.86)	0.1044	-16.99 (-91.31; 57.33)	0.6534
	11	13	219.1 (29.82)	-31.0 (27.57)	0.2612	14	219.5 (28.56)	-17.4 (26.36)	0.5084	13.57 (-61.43; 88.57)	0.7222
	12	13	205.0 (29.82)	-45.1 (27.57)	0.1024	14	219.6 (28.56)	-17.3 (26.36)	0.5119	27.83 (-47.17; 102.83)	0.4661
	13	12	217.2 (30.39)	-32.9 (28.19)	0.2433	12	249.2 (29.66)	12.30 (27.55)	0.6555	45.24 (-32.26; 122.73)	0.2518
	14	10	183.3 (31.83)	-66.9 (29.73)	0.0250	12	252.3 (29.66)	15.37 (27.55)	0.5773	82.26 (2.56; 161.95)	0.0431
	15	8	184.6 (33.86)	-65.6 (31.89)	0.0404	10	208.5 (31.12)	-28.4 (29.12)	0.3297	37.18 (-47.74; 122.09)	0.3899
	16	6	175.9 (36.99)	-74.3 (35.19)	0.0354	9	169.1 (32.06)	-67.8 (30.12)	0.0250	6.49 (-84.58; 97.57)	0.8886

IBS-SSS, Irritable Bowel Syndrome Symptom Severity Score.
