# Supplementary material for: Multistrain Probiotics Plus Vitamin D Improve Gut Barrier Function and Gut Microbiota Composition in Irritable Bowel Syndrome Without Constipation: Results from a Double-Blind, Randomized, Placebo-Controlled Trial
Source: Nutrients. 2025 May 18;17(10):1708. doi: 10.3390/nu17101708 (PMC12114473; doi:10.3390/nu17101708)
Supplement: Supplementary file 1 [file nutrients-17-01708-s001.zip › Ottabac_Supplementary Table S3.pdf]

Supplementary Table S3. Marginal mean of abdominal pain, bloating, and Irritable Bowel Syndrome Symptom Severity Score (IBS-SSS) at each week and variation from baseline by group, per-protocol.

		Placebo				Active					
				change from baseline				change from baseline		Active vs Placebo	
Parameter	Week	n	mean (se)	mean (se)	pvalue	n	mean (se)	mean (se)	pvalue	Mean Diff of variation Active-Placebo (95% CI)	pvalue
Abdominal pain intensity	0	14	39.14 (7.62)			12	37.75 (8.23)				
	1	13	32.38 (7.87)	-6.76 (8.64)	0.4346	12	27.50 (8.23)	-10.3 (9.09)	0.2603	-3.49 (-28.16; 21.18)	0.7808
	2	13	32.38 (7.87)	-6.76 (8.64)	0.4346	12	36.17 (8.23)	-1.58 (9.09)	0.8618	5.17 (-19.49; 29.84)	0.6801
	3	13	28.00 (7.87)	-11.1 (8.64)	0.1981	12	24.83 (8.23)	-12.9 (9.09)	0.1562	-1.77 (-26.44; 22.9)	0.8876
	4	13	28.85 (7.87)	-10.3 (8.64)	0.2342	12	19.33 (8.23)	-18.4 (9.09)	0.0435	-8.12 (-32.79; 16.55)	0.5177
	5	13	43.47 (8.32)	4.33 (9.05)	0.6331	12	23.08 (8.49)	-14.7 (9.32)	0.1166	-19 (-44.56; 6.57)	0.1448
	6	13	29.62 (7.87)	-9.53 (8.64)	0.2710	12	18.99 (8.49)	-18.8 (9.32)	0.0450	-9.24 (-34.24; 15.77)	0.4680
	7	13	33.38 (7.87)	-5.76 (8.64)	0.5056	12	32.44 (8.49)	-5.31 (9.32)	0.5696	0.45 (-24.55; 25.45)	0.9718
	8	13	23.92 (7.87)	-15.2 (8.64)	0.0791	12	22.35 (8.49)	-15.4 (9.32)	0.0996	-0.18 (-25.18; 24.82)	0.9887
	9	13	36.18 (8.08)	-2.96 (8.83)	0.7378	12	25.62 (8.49)	-12.1 (9.32)	0.1944	-9.17 (-34.43; 16.1)	0.4758
	10	13	35.62 (7.87)	-3.53 (8.64)	0.6833	12	31.71 (8.49)	-6.04 (9.32)	0.5179	-2.51 (-27.51; 22.5)	0.8437
	11	13	35.31 (7.87)	-3.84 (8.64)	0.6574	11	23.40 (8.49)	-14.4 (9.32)	0.1247	-10.52 (-35.52; 14.49)	0.4085
	12	13	34.81 (7.87)	-4.34 (8.64)	0.6162	11	26.99 (8.49)	-10.8 (9.32)	0.2493	-6.43 (-31.43; 18.58)	0.6134
	13	12	36.55 (8.08)	-2.60 (8.83)	0.7691	10	34.82 (8.77)	-2.93 (9.58)	0.7601	-0.33 (-25.96; 25.3)	0.9797
	14	10	28.78 (8.61)	-10.4 (9.32)	0.2668	10	28.32 (9.10)	-9.43 (9.88)	0.3406	0.94 (-25.78; 27.65)	0.9451
	15	8	32.56 (9.34)	-6.58 (10.00)	0.5110	9	24.67 (9.10)	-13.1 (9.88)	0.1866	-6.5 (-34.15; 21.15)	0.6441
	16	6	28.32 (10.44)	-10.8 (11.04)	0.3277	9	22.34 (9.10)	-15.4 (9.88)	0.1198	-4.59 (-33.73; 24.55)	0.7567
Abdominal pain frequency	0	14	37.14 (8.71)			12	35.00 (9.41)				
	1	13	28.92 (8.94)	-8.22 (8.31)	0.3232	12	30.83 (9.41)	-4.17 (8.71)	0.6323	4.05 (-19.63; 27.73)	0.7366
	2	13	28.92 (8.94)	-8.22 (8.31)	0.3232	12	37.50 (9.41)	2.50 (8.71)	0.7746	10.72 (-12.96; 34.39)	0.3739

	3	13	33.53 (8.94)	-3.61 (8.31)	0.6645	12	31.67 (9.41)	-3.34 (8.71)	0.7018	0.27 (-23.41; 23.95)	0.9822
	4	13	29.69 (8.94)	-7.45 (8.31)	0.3704	12	21.67 (9.41)	-13.3 (8.71)	0.1265	-5.88 (-29.56; 17.79)	0.6252
	5	13	40.53 (9.11)	3.39 (8.49)	0.6899	12	24.17 (9.41)	-10.8 (8.71)	0.2141	-14.23 (-38.16; 9.7)	0.2429
	6	13	27.38 (8.94)	-9.76 (8.31)	0.2410	12	21.67 (9.41)	-13.3 (8.71)	0.1265	-3.58 (-27.25; 20.1)	0.7665
	7	13	32.00 (8.94)	-5.15 (8.31)	0.5362	12	29.17 (9.41)	-5.84 (8.71)	0.5031	-0.69 (-24.37; 22.98)	0.9542
	8	13	25.08 (8.94)	-12.1 (8.31)	0.1475	12	33.82 (9.61)	-1.19 (8.92)	0.8941	10.88 (-13.11; 34.86)	0.3730
	9	13	32.00 (8.94)	-5.14 (8.31)	0.5365	12	32.91 (9.61)	-2.10 (8.92)	0.8143	3.04 (-20.94; 27.03)	0.8030
	10	13	34.30 (8.94)	-2.84 (8.31)	0.7330	12	30.83 (9.41)	-4.17 (8.71)	0.6323	-1.33 (-25.01; 22.34)	0.9119
	11	13	32.00 (8.94)	-5.14 (8.31)	0.5365	11	28.36 (9.61)	-6.64 (8.92)	0.4571	-1.5 (-25.48; 22.48)	0.9021
	12	13	30.46 (8.94)	-6.68 (8.31)	0.4220	11	23.82 (9.61)	-11.2 (8.92)	0.2108	-4.51 (-28.49; 19.48)	0.7118
	13	12	32.42 (9.11)	-4.72 (8.50)	0.5792	10	31.85 (9.84)	-3.16 (9.17)	0.7309	1.56 (-23.03; 26.14)	0.9008
	14	10	26.86 (9.55)	-10.3 (8.96)	0.2520	10	36.85 (9.84)	1.85 (9.17)	0.8402	12.13 (-13.08; 37.34)	0.3446
	15	8	31.49 (10.16)	-5.65 (9.61)	0.5568	9	25.06 (10.11)	-9.94 (9.46)	0.2940	-4.29 (-30.81; 22.23)	0.7506
	16	6	23.22 (11.10)	-13.9 (10.60)	0.1900	9	22.90 (10.11)	-12.1 (9.46)	0.2014	1.82 (-26.13; 29.77)	0.8983
Abdominal distension	0	14	44.57 (7.71)			12	37.93 (8.51)				
	1	13	39.86 (8.09)	-4.71 (7.75)	0.5441	12	43.39 (8.51)	5.45 (8.30)	0.5118	10.16 (-12.19; 32.51)	0.3717
	2	13	42.61 (8.09)	-1.96 (7.75)	0.8007	12	35.25 (8.33)	-2.69 (8.14)	0.7418	-0.73 (-22.84; 21.39)	0.9485
	3	13	33.44 (7.92)	-11.1 (7.58)	0.1431	12	31.83 (8.33)	-6.10 (8.14)	0.4540	5.02 (-16.86; 26.91)	0.6518
	4	13	30.75 (7.92)	-13.8 (7.58)	0.0692	12	27.08 (8.33)	-10.9 (8.14)	0.1834	2.97 (-18.92; 24.85)	0.7899
	5	13	41.79 (8.27)	-2.79 (7.95)	0.7262	12	23.25 (8.33)	-14.7 (8.14)	0.0722	-11.9 (-34.28; 10.48)	0.2963
	6	13	32.18 (8.08)	-12.4 (7.75)	0.1107	12	29.05 (8.52)	-8.88 (8.35)	0.2882	3.51 (-18.89; 25.92)	0.7578
	7	13	33.67 (7.92)	-10.9 (7.58)	0.1516	12	34.14 (8.52)	-3.79 (8.35)	0.6501	7.11 (-15.07; 29.29)	0.5289
	8	13	23.06 (7.92)	-21.5 (7.58)	0.0048	12	26.78 (8.52)	-11.2 (8.35)	0.1824	10.36 (-11.82; 32.54)	0.3589
	9	13	37.20 (8.09)	-7.37 (7.75)	0.3425	12	41.99 (8.74)	4.06 (8.54)	0.6351	11.43 (-11.26; 34.11)	0.3225
	10	13	39.44 (7.92)	-5.13 (7.58)	0.4991	12	37.10 (8.74)	-0.83 (8.57)	0.9225	4.3 (-18.21; 26.8)	0.7075
	11	13	40.14 (7.92)	-4.44 (7.58)	0.5589	11	33.32 (8.52)	-4.61 (8.35)	0.5813	-0.17 (-22.35; 22.01)	0.9879
	12	13	34.98 (7.92)	-9.59 (7.58)	0.2068	11	37.87 (8.52)	-0.06 (8.35)	0.9941	9.53 (-12.65; 31.71)	0.3987
	13	12	35.48 (8.08)	-9.09 (7.75)	0.2417	10	42.62 (8.74)	4.68 (8.57)	0.5850	13.78 (-8.95; 36.51)	0.2340

	14	10	29.29 (8.50)	-15.3 (8.18)	0.0626	10	55.42 (8.74)	17.49 (8.57)	0.0421	32.77 (9.47; 56.07)	0.0060
	15	8	23.08 (9.07)	-21.5 (8.78)	0.0148	9	38.95 (8.99)	1.02 (8.83)	0.9084	22.51 (-1.98; 47)	0.0714
	16	6	30.96 (9.95)	-13.6 (9.68)	0.1609	9	22.34 (8.99)	-15.6 (8.83)	0.0784	-1.98 (-27.76; 23.8)	0.8798
Satisfaction of bowel habits	0	14	63.71 (4.75)			12	65.25 (5.13)				
	1	13	59.70 (4.88)	-4.01 (4.87)	0.4111	12	57.50 (5.13)	-7.75 (5.12)	0.1309	-3.74 (-17.64; 10.16)	0.5970
	2	13	58.86 (4.88)	-4.86 (4.87)	0.3197	12	60.75 (5.13)	-4.50 (5.12)	0.3799	0.36 (-13.55; 14.26)	0.9598
	3	13	54.17 (4.88)	-9.55 (4.87)	0.0509	12	58.50 (5.13)	-6.75 (5.12)	0.1881	2.8 (-11.1; 16.7)	0.6924
	4	13	56.24 (4.88)	-7.47 (4.87)	0.1262	12	57.67 (5.13)	-7.58 (5.12)	0.1393	-0.11 (-14.01; 13.79)	0.9874
	5	13	62.62 (4.99)	-1.09 (4.98)	0.8265	12	57.63 (5.40)	-7.62 (5.39)	0.1585	-6.53 (-20.98; 7.91)	0.3745
	6	13	53.78 (4.88)	-9.93 (4.87)	0.0423	12	56.52 (5.26)	-8.73 (5.25)	0.0974	1.2 (-12.89; 15.3)	0.8667
	7	13	58.55 (4.88)	-5.16 (4.87)	0.2901	12	59.36 (5.41)	-5.89 (5.40)	0.2758	-0.73 (-15.03; 13.58)	0.9204
	8	13	56.01 (4.88)	-7.70 (4.87)	0.1149	12	54.29 (5.26)	-11.0 (5.25)	0.0377	-3.25 (-17.35; 10.84)	0.6500
	9	13	61.82 (4.88)	-1.89 (4.87)	0.6976	12	54.61 (5.26)	-10.6 (5.25)	0.0436	-8.74 (-22.84; 5.35)	0.2232
	10	13	58.70 (4.88)	-5.01 (4.87)	0.3047	12	52.46 (5.41)	-12.8 (5.40)	0.0184	-7.78 (-22.09; 6.52)	0.2854
	11	13	57.24 (4.99)	-6.47 (4.98)	0.1947	11	56.06 (5.41)	-9.19 (5.40)	0.0895	-2.72 (-17.17; 11.73)	0.7115
	12	13	53.55 (4.88)	-10.2 (4.87)	0.0378	11	53.76 (5.41)	-11.5 (5.40)	0.0340	-1.33 (-15.63; 12.98)	0.8553
	13	12	54.87 (4.99)	-8.84 (4.98)	0.0768	10	62.08 (5.40)	-3.17 (5.40)	0.5567	5.67 (-8.78; 20.12)	0.4406
	14	10	52.23 (5.44)	-11.5 (5.43)	0.0351	10	57.78 (5.40)	-7.47 (5.40)	0.1670	4.02 (-11.04; 19.08)	0.6001
	15	8	48.83 (5.91)	-14.9 (5.90)	0.0121	9	58.96 (5.58)	-6.29 (5.57)	0.2593	8.59 (-7.36; 24.55)	0.2903
	16	6	52.24 (6.66)	-11.5 (6.66)	0.0857	9	50.14 (5.78)	-15.1 (5.77)	0.0093	-3.64 (-20.97; 13.7)	0.6801
Interference on life	0	14	65.50 (5.02)			12	61.13 (5.53)				
	1	13	64.06 (5.14)	-1.43 (4.75)	0.7625	12	56.26 (5.42)	-4.87 (5.10)	0.3399	-3.74 (-17.64; 10.16)	0.5970
	2	13	58.56 (5.49)	-6.94 (5.12)	0.1762	12	60.67 (5.68)	-0.46 (5.38)	0.9319	0.36 (-13.55; 14.26)	0.9598
	4	13	55.29 (5.36)	-10.2 (4.98)	0.0411	12	54.50 (5.42)	-6.63 (5.10)	0.1942	-0.11 (-14.01; 13.79)	0.9874
	5	13	61.07 (5.36)	-4.43 (4.97)	0.3739	12	51.46 (5.68)	-9.67 (5.38)	0.0733	-6.53 (-20.98; 7.91)	0.3745
	6	13	58.76 (5.14)	-6.74 (4.75)	0.1567	12	56.99 (5.55)	-4.14 (5.24)	0.4299	1.2 (-12.89; 15.3)	0.8667
	7	13	52.76 (5.14)	-12.7 (4.75)	0.0077	12	51.72 (5.55)	-9.42 (5.24)	0.0735	-0.73 (-15.03; 13.58)	0.9204

	8	13	57.57 (5.14)	-7.93 (4.75)	0.0958	12	56.17 (5.55)	-4.96 (5.24)	0.3447	-3.25 (-17.35; 10.84)	0.6500
	9	13	59.18 (5.14)	-6.31 (4.75)	0.1843	12	51.31 (5.55)	-9.82 (5.24)	0.0619	-8.74 (-22.84; 5.35)	0.2232
	10	13	56.76 (5.14)	-8.74 (4.75)	0.0666	12	47.99 (5.55)	-13.1 (5.24)	0.0127	-7.78 (-22.09; 6.52)	0.2854
	11	13	59.38 (5.14)	-6.12 (4.75)	0.1980	11	49.81 (5.55)	-11.3 (5.24)	0.0315	-2.72 (-17.17; 11.73)	0.7115
	12	13	54.46 (5.24)	-11.0 (4.85)	0.0237	11	52.32 (5.68)	-8.81 (5.36)	0.1014	-1.33 (-15.63; 12.98)	0.8553
	13	12	57.74 (5.24)	-7.76 (4.85)	0.1108	10	58.16 (5.68)	-2.97 (5.38)	0.5818	5.67 (-8.78; 20.12)	0.4406
	14	10	55.87 (5.65)	-9.62 (5.29)	0.0700	10	62.42 (5.68)	1.29 (5.38)	0.8114	4.02 (-11.04; 19.08)	0.6001
	15	8	59.98 (6.08)	-5.52 (5.75)	0.3378	9	53.66 (5.83)	-7.47 (5.52)	0.1776	8.59 (-7.36; 24.55)	0.2903
	16	6	56.21 (6.79)	-9.29 (6.49)	0.1532	9	44.87 (6.02)	-16.3 (5.72)	0.0048	-3.64 (-20.97; 13.7)	0.6801
IBS-SSS	0	14	250.0 (28.34)			12	230.4 (30.61)				
	1	13	221.7 (29.10)	-28.3 (27.71)	0.3077	12	211.7 (30.61)	-18.7 (29.08)	0.5198	9.57 (-69.44; 88.59)	0.8118
	2	13	206.0 (29.10)	-44.1 (27.71)	0.1127	12	219.1 (30.61)	-11.3 (29.08)	0.6973	32.76 (-46.26; 111.78)	0.4154
	3	13	149.5 (29.10)	-101 (27.71)	0.0003	12	146.8 (30.61)	-83.6 (29.08)	0.0043	16.97 (-62.05; 95.99)	0.6729
	4	13	190.7 (29.10)	-59.3 (27.71)	0.0331	12	180.2 (30.61)	-50.2 (29.08)	0.0855	9.16 (-69.86; 88.17)	0.8198
	5	13	242.0 (29.69)	-8.04 (28.33)	0.7767	12	158.5 (30.61)	-71.9 (29.08)	0.0139	-63.88 (-143.75; 15.98)	0.1165
	6	13	199.8 (29.10)	-50.2 (27.71)	0.0708	12	168.7 (30.61)	-61.7 (29.08)	0.0347	-11.42 (-90.44; 67.6)	0.7764
	7	13	210.7 (29.10)	-39.4 (27.71)	0.1562	12	186.9 (30.61)	-43.5 (29.08)	0.1357	-4.1 (-83.12; 74.92)	0.9188
	8	13	185.9 (29.10)	-64.1 (27.71)	0.0213	12	187.2 (31.30)	-43.2 (29.80)	0.1478	20.88 (-59.17; 100.93)	0.6082
	9	13	220.9 (29.10)	-29.2 (27.71)	0.2936	12	196.2 (31.30)	-34.2 (29.80)	0.2514	-5.08 (-85.13; 74.97)	0.9007
	10	13	225.1 (29.10)	-24.9 (27.71)	0.3691	12	180.6 (30.61)	-49.8 (29.08)	0.0876	-24.89 (-103.91; 54.12)	0.5359
	11	13	219.1 (29.10)	-30.9 (27.71)	0.2653	11	180.5 (31.30)	-49.9 (29.80)	0.0951	-18.95 (-99; 61.1)	0.6418
	12	13	205.0 (29.10)	-45.0 (27.71)	0.1051	11	181.8 (31.30)	-48.6 (29.80)	0.1035	-3.61 (-83.65; 76.44)	0.9295
	13	12	217.4 (29.69)	-32.6 (28.34)	0.2502	10	223.1 (32.08)	-7.32 (30.62)	0.8112	25.32 (-56.74; 107.38)	0.5443
	14	10	183.3 (31.17)	-66.8 (29.89)	0.0261	10	232.5 (32.08)	2.08 (30.62)	0.9459	68.85 (-15.31; 153.01)	0.1085
	15	8	184.8 (33.27)	-65.3 (32.06)	0.0426	9	193.5 (33.00)	-36.9 (31.58)	0.2435	28.37 (-60.15; 116.9)	0.5288
	16	6	176.0 (36.48)	-74.0 (35.38)	0.0372	9	145.5 (33.00)	-84.9 (31.58)	0.0075	-10.91 (-104.2; 82.39)	0.8183

IBS-SSS, Irritable Bowel Syndrome Symptom Severity Score.
