# Supplementary material for: Multistrain Probiotics Plus Vitamin D Improve Gut Barrier Function and Gut Microbiota Composition in Irritable Bowel Syndrome Without Constipation: Results from a Double-Blind, Randomized, Placebo-Controlled Trial
Source: Nutrients. 2025 May 18;17(10):1708. doi: 10.3390/nu17101708 (PMC12114473; doi:10.3390/nu17101708)
Supplement: Supplementary file 1 [file nutrients-17-01708-s001.zip › Supplementary Figures Legends.pdf]

Supplementary Figures Legends

Supplementary Figure S1. Microbiota comparison between the time points per treatment by Wilcoxon test. The figure displays the Wilcoxon results with the median values for all comparisons between time points (V2, V4, V5) for each treatment (Active or Placebo). The medians are labeled in blue or red, indicating high or low bacterial abundance, respectively. The taxonomy for all variables is provided in the following format: p, phylum; c, class; o, order; f, family; g, genus; s, species; ASV, amplicon sequence variant. The p__Actinobacteria.c__Actinobacteria.o__Bifidobacteriales.f__Bifidobacteriaceae.g__Bifidobacterium.ASV_ 1629 and the p__Firmicutes.c__Bacilli.o__Lactobacillales.f__Streptococcaceae.g__Streptococcus.s__.ASV_2914 were manually checked using BLAST, matching Bifidobacterium animalis subsp. lactis and Streptococcus thermophilus, respectively.

Supplementary Figure S2. SCFAs comparison between the time points per treatment by Wilcoxon or Ttest. The figure displays the Wilcoxon or Ttest (in dependence on the Shapiro francia test of normality of the data) results with the median values for all comparisons between time points (V2, V4, V5) for each treatment (Active or Placebo). The medians or means (in dependence on the Shapiro francia test of normality of the data) are labeled in blue or red, indicating high or low SCFAs abundance, respectively. The SCFAs under analyses are: Lac, lactic acid; AA, acetic acid; SA, succinic acid, PA, propionic acid; BA, butyric acid; iBA iso-butyric acid; iVA, iso-valeric acid; VA, Valeric acid. In bold the significance with $P < 0.05$ and in blue the results from the Ttest.

Supplementary Figure S3. Microbiota comparison between the treatment on time V4 by Mann-Whitney test. The figure displays the Mann-Whitney results with the median values for all comparisons between treatments (Active, Placebo) at time V4 . The medians are labeled in blue or red, indicating high or low bacterial abundance, respectively. The taxonomy for all variables is provided in the following format: p, phylum; c, class; o, order; f, family; g, genus; s, species; ASV, amplicon sequence variant. The p__Actinobacteria.c__Actinobacteria.o__Bifidobacteriales.f__Bifidobacteriaceae.g__Bifidobacterium. ASV_ 1629 was manually checked using BLAST, matching *Bifidobacterium animalis* subsp. l*actis*. The number of subjects took in consideration for the analyses was 26 (14 Placebo, 12 Active).

Supplementary Figure S4. Kendall correlation at baseline. This figure presents the correlations determined by the Kendall test between clinical variables, SCFAs, and alpha diversity indexes with bacteria that were found to differ significantly between time points V2 and V4 in the two treatments, as determined by the Wilcoxon test. The median levels of specific bacteria at V2 and V4 are indicated by red and blue, respectively, with red representing a higher level and blue representing a lower level. In the correlation matrix, red and green colors indicate negative and positive correlations, respectively. The numbers represent the p-values, where a negative correlation is indicated by a *p-value* < 0. The table includes only those bacteria that have at least one significant correlation with the listed variables.
