# Supplementary figures and images for: Multistrain Probiotics Plus Vitamin D Improve Gut Barrier Function and Gut Microbiota Composition in Irritable Bowel Syndrome Without Constipation: Results from a Double-Blind, Randomized, Placebo-Controlled Trial
Source: Nutrients. 2025 May 18;17(10):1708. doi: 10.3390/nu17101708 (PMC12114473; doi:10.3390/nu17101708)

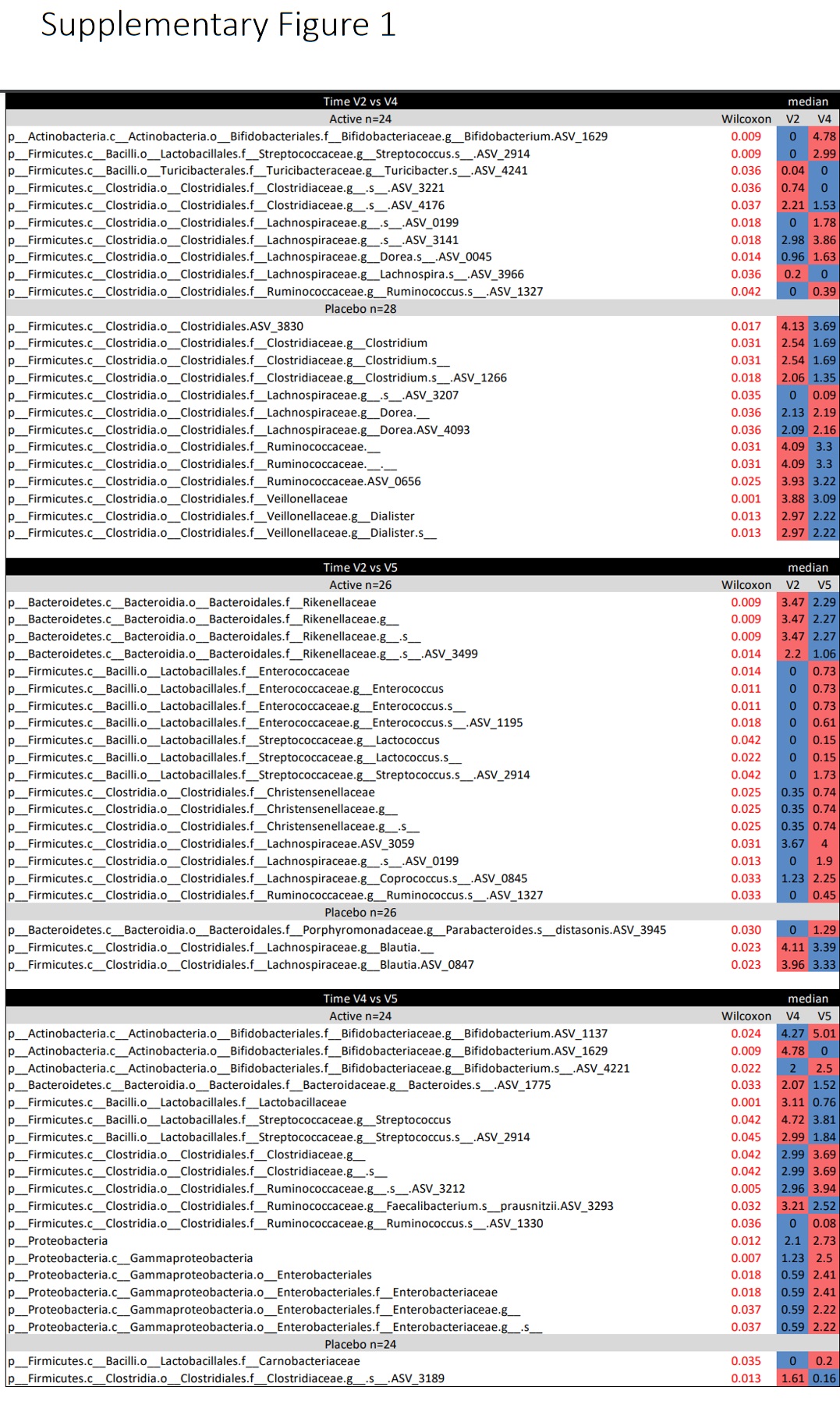

Supplement: Supplementary file 1 [file nutrients-17-01708-s001.zip › supplementary figure S1.jpg]

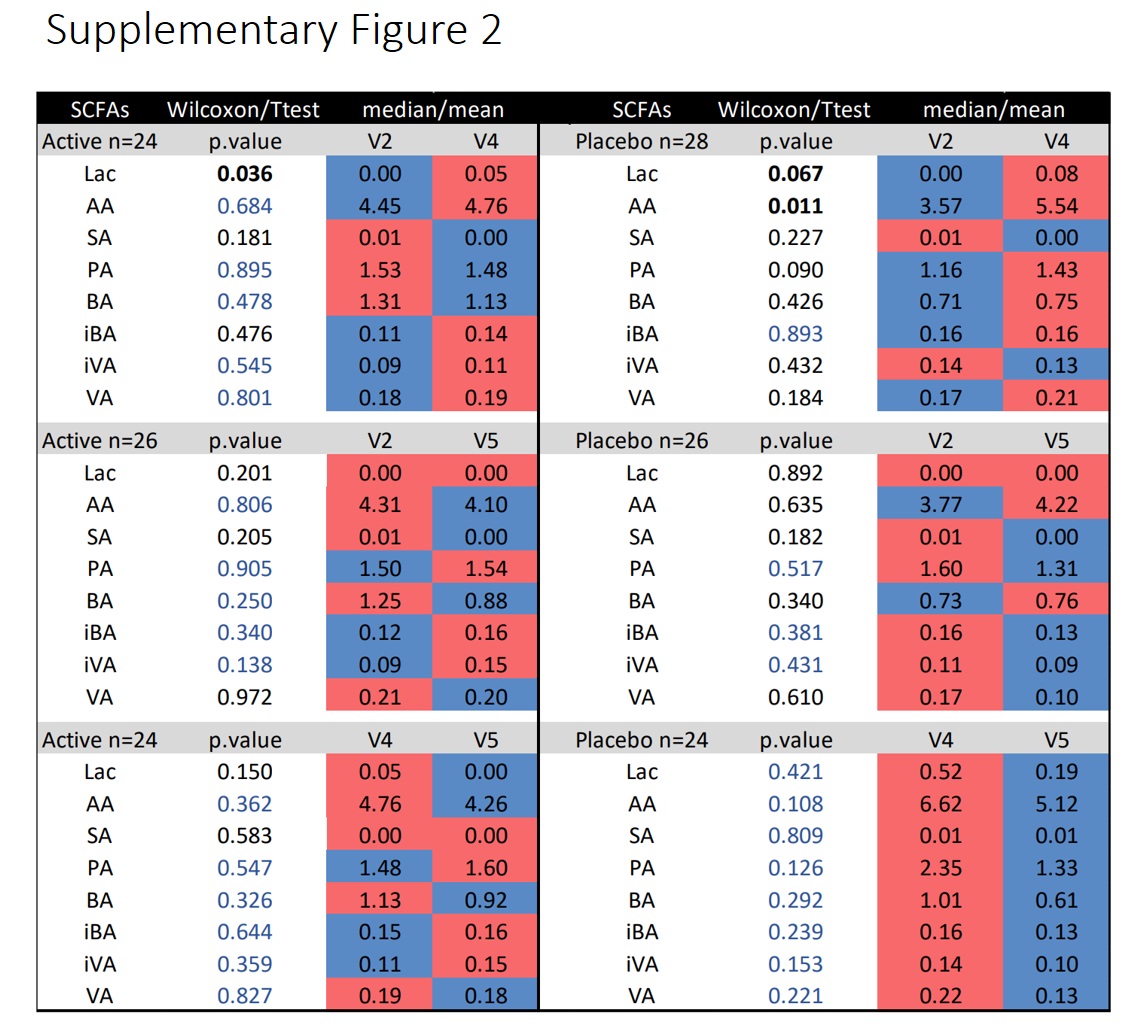

Supplement: Supplementary file 1 [file nutrients-17-01708-s001.zip › supplementary figure S2.jpg]

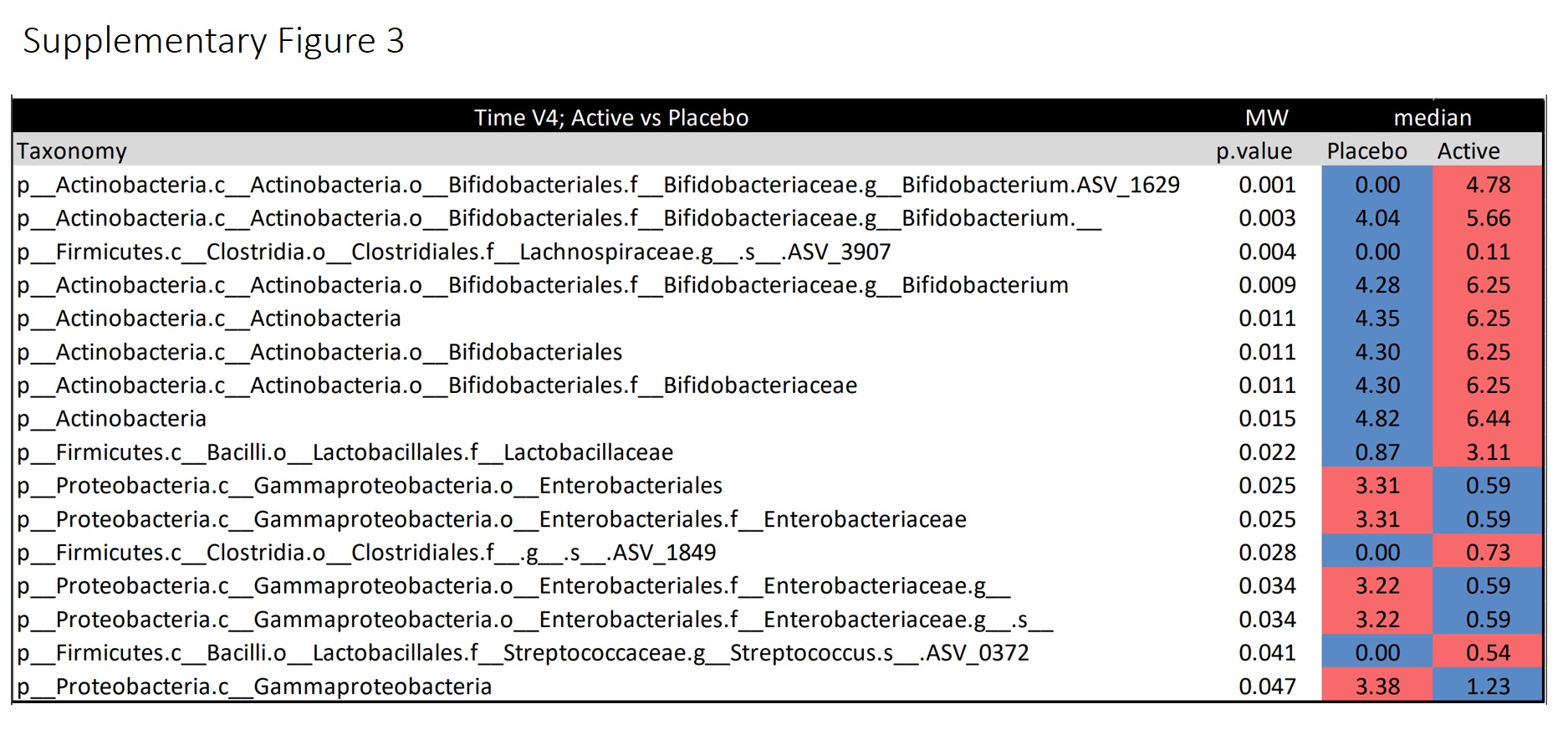

Supplement: Supplementary file 1 [file nutrients-17-01708-s001.zip › supplementary figure S3.jpg]

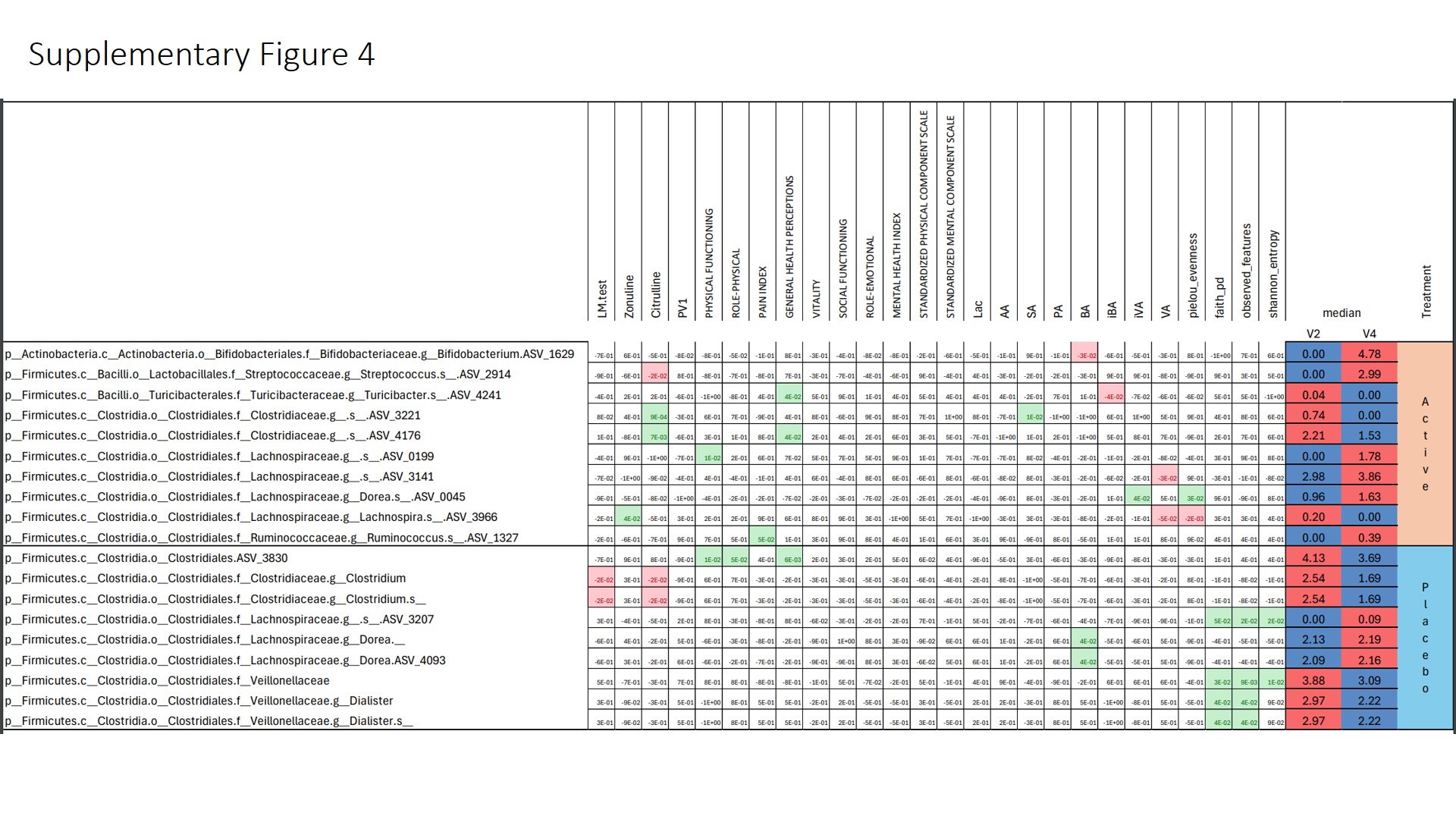

Supplement: Supplementary file 1 [file nutrients-17-01708-s001.zip › supplementary figure S4.jpg]
